# Social determinants and exposure to intimate partner violence in women with severe acute maternal morbidity in the intensive care unit: a systematic review

**DOI:** 10.1186/s12884-023-05927-5

**Published:** 2023-09-12

**Authors:** Beatriz Paulina Ayala Quintanilla, Angela Taft, Susan McDonald, Wendy Pollock, Joel Christian Roque Henriquez

**Affiliations:** 1 School of Nursing and Midwifery, The Judith Lumley Centre, La Trobe University, Plenty Road & Kingsbury Drive, Level 3, George Singer Building, Bundoora, Melbourne, VIC Australia; 2 Universidad de San Martin de Porres, La Molina, Lima, Peru; 3 Nursing and Midwifery, Monash University, Melbourne, VIC Australia

**Keywords:** Pregnancy, Domestic violence, Intimate partner violence, Social determinants of health, Intensive care units, Systematic review

## Abstract

**Background:**

Studying severe acute maternal morbidity in the intensive care unit improves our understanding of potential factors affecting maternal health.

**Aim:**

To review evidence on maternal exposure to intimate partner violence and social determinants of health in women with severe acute maternal morbidity in the intensive care unit.

**Methods:**

The protocol for this review was registered in PROSPERO (registration number CRD42016037492). A systematic search was performed in MEDLINE, CINAHL, ProQuest, LILACS and SciELO using the search terms “intensive care unit”, “intensive care”, “critical care” and “critically ill” in combination with “intimate partner violence”, “social determinants of health”, “severe acute maternal morbidity”, pregnancy, postpartum and other similar terms. Eligible studies were (i) quantitative, (ii) published in English and Spanish, (iii) from 2000 to 2021, (iv) with data related to intimate partner violence and/or social determinants of health, and (v) investigating severe acute maternal morbidity (maternity patients treated in the intensive care unit during pregnancy, childbirth or within 42 days of pregnancy termination). Of 52,866 studies initially identified, 1087 full texts were assessed and 156 studies included. Studies were independently assessed by two reviewers for screening, revision, quality assessment and abstracted data. Studies were categorised into high/middle/low-income countries and summarised data were presented using a narrative description, due to heterogenic data as: (i) exposure to intimate partner violence and (ii) social determinants of health.

**Results:**

One study assessed intimate partner violence among mothers with severe acute maternal morbidity in the intensive care unit and found that women exposed to intimate partner violence before and during pregnancy had a nearly four-fold risk of severe acute maternal morbidity requiring ICU admission. Few social determinants of health other than age were reported in most studies.

**Conclusion:**

This review identified a significant gap in knowledge concerning intimate partner violence and social determinants of health in women with severe acute maternal morbidity in the intensive care unit, which is essential to better understand the complete picture of the maternal morbidity spectrum and reduce maternal mortality.

**Supplementary Information:**

The online version contains supplementary material available at 10.1186/s12884-023-05927-5.

## Background

Maternal mortality and intimate partner violence (IPV) are potentially preventable public health issues. Globally, one in three women have experienced lifetime prevalence of IPV [1] and during pregnancy the IPV rate varies from 1 to 49% [2]. In high-income countries, there has been increased recognition of an association between IPV and maternal mortality [3]; however, maternal mortality is the tip of the iceberg, with the unexposed base formed by maternal morbidity cases [4]. This includes women with severe acute maternal morbidity (SAMM), who are obstetric patients who nearly died but have survived [5].

Currently, there is no accepted operational definition for SAMM, and it has been evaluated using diverse criteria across studies [5–7]. The World Health Organization (WHO) developed a tool including organ system dysfunction parameters for defining SAMM [5], however, some investigators consider that the application of this tool may be too complex in both low and high-income countries [8]. ICU admission has a high sensitivity and specificity for identifying most very sick women (the near miss maternal patients) [6, 9]. Thus, obstetric patients with SAMM in the intensive care unit (ICU) represent the most critically ill obstetric patients and can be considered as an alternative marker for SAMM [6, 7]. The ICU SAMM admission rate ranges from 0.04 to 4.54%, and the main causes are hypertensive disorders of pregnancy, obstetric haemorrhage, and sepsis [10–12].

According to the maternal morbidity framework [13], it is important to consider not only clinical and biological aspects, but also other factors, among which IPV and social determinants of health are included, in order to capture everything that matters to better understand maternal morbidity [13]. Social determinants of health are non-medical factors; conditions in which people are born, grow, work, live, and age, and the wider set of forces and systems shaping the conditions of daily life [14, 15]. Studying SAMM provides data to complement the review of maternal deaths [16–19], and makes it possible to investigate underlying factors that may contribute to making improvements in maternity care [5, 18].

The association of violence against women and maternal death has been highlighted in recent years (3), and some studies have shown adverse outcomes of IPV on women’s health including during pregnancy [20, 21], when women could be more vulnerable to IPV [22]. More knowledge is needed about IPV and social determinants of health in women with SAMM to better understand factors relevant to the maternal morbidity framework [13]. Therefore, this study aimed to review available evidence of exposure to IPV as well as social determinants of health in maternity patients with SAMM in the ICU.

## Methods

This systematic review was registered (CRD42016037492) in PROSPERO (International prospective register of systematic reviews) and followed the PRISMA (Preferred Reporting Items for Systematic Reviews and Meta-Analyses) Statement [23]. Detailed design and methodology were published in a study protocol [24].

### Search strategy

A systematic search was performed in December 2021 in MEDLINE, ProQuest, CINAHL, Latin American and Caribbean Health Science Information Database (LILACS) and SciELO (Scientific Electronic Library Online) between 1st January 2000 and 15th December 2021. The following subject heading and/or free text words were used: intensive care unit, intensive care, critical care and critically ill in combination with the following terms: IPV, social determinants of health, severe acute maternal morbidity, pregnancy, postpartum, etc. Other relevant terms are described in detail in Supplementary Appendix S1. Hand searching was also conducted.

The starting point of the year 2000 was considered appropriate because associations between social inequalities and vulnerabilities, IPV and maternal death were first described in the 1997–1999 UK mortality review [25]. We expected that literature from 2000 would better recognise these associations to address the lack of data on social determinants of health and IPV in studies investigating severe maternal morbidity [24].

### Eligibility criteria

The inclusion criteria were: (i) experimental and observational studies; (ii) women admitted to an ICU during pregnancy, childbirth or within 42 days of pregnancy termination; (iii) the total population of maternity patients managed in an ICU (and not a sub-set e.g., preeclampsia); (iv) studies written in English or Spanish; (v) published between the period January 2000 and December 2021; and (vi) studies with data related to IPV and/or social determinants of health.

The exclusion criteria were (i) any qualitative investigations, theses, study protocols, congress abstracts or reviews, case reports, editorials, opinions, letters, and weekly reports; (ii) studies evaluating specific condition(s) or subgroup of patients managed in the ICU during pregnancy, childbirth or within 42 days of pregnancy termination; and (iii) duplicate studies using the same data published in different journals (in this case the most recent or relevant publication was selected).

### Study selection

The screening of titles and/or abstracts and/or full texts of studies were assessed independently by two reviewers (BPAQ and SMc or WP). The first reviewer retrieved full texts of potentially eligible studies. After that, the assessment and decision for inclusion of studies were performed independently by the reviewers, two for studies written in English (BPAQ and SMc or WP), and two for studies in Spanish (BPAQ and JR). Disagreements were resolved by discussion and consensus between the reviewers, and consultation with another reviewer was not needed.

### Data extraction

Three reviewers (BPAQ, WP and JR) independently extracted data [24] using a standardised data extraction form. We extracted data on country, language, study design, study period, setting, sample size, name of journal, IPV, social determinants of health and substance use. Data on social determinants of health included age, race, ethnicity and migration status, marital status, place of residence, education, employment and income, socio-economic status, and health insurance status. Data on substance abuse comprised alcohol consumption, smoking and drug abuse.

Data were analysed and presented using narrative description. When data were sufficient, findings were explained using the World Bank’s classification of countries [26] and presented in the following main categories (i) exposure to IPV; and (ii) social determinants of health. Data on age were meta-analysed and were shown as means (± standard deviation). All authors discussed and revised the synthesised data and extracted data.

### Risk of bias and quality appraisal of the studies

Bias and quality were assessed using the Critical Appraisal Skills Programme (CASP) checklist [27]. CASP is a tool which systematically assesses the trustworthiness, relevance, and results of the studies. This process was performed independently by two reviewers for studies in English (BPAQ and SMc or WP), and two for studies in Spanish (BPAQ and JR).

## Results

**Fig. 1:**
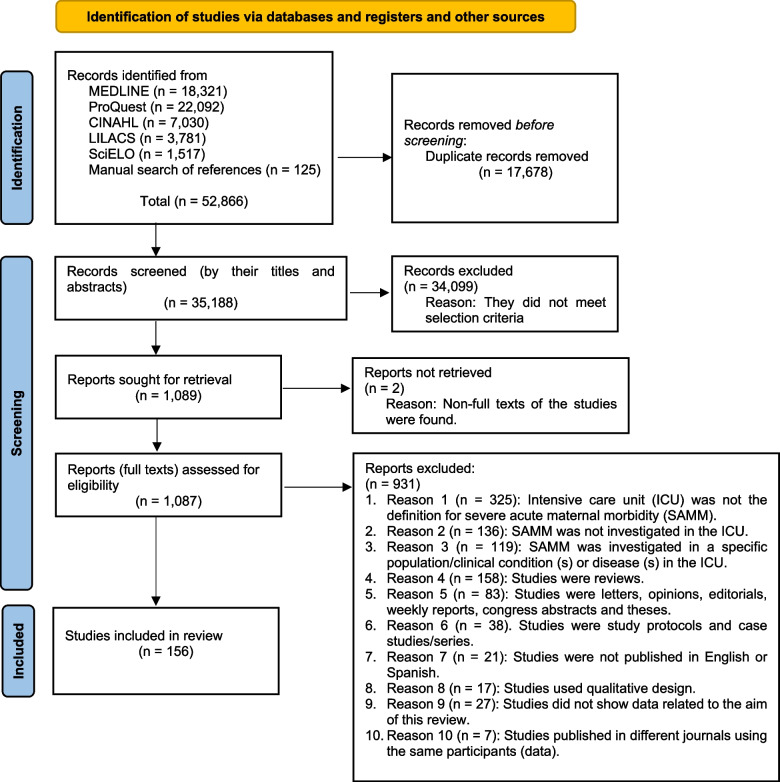
PRISMA flow diagram of study selection. *Adapted from*: Page MJ, McKenzie JE, Bossuyt PM, Boutron I, Hoffmann TC, Mulrow CD, et al. The PRISMA 2020 statement: an updated guideline for reporting systematic reviews. BMJ 2021;372:n71. doi: 10.1136/bmj.n71. For more information, visit: http://www.prisma-statement.org/

We initially identified 52,866 studies from the five databases and manual search of references, of which 35,188 titles and abstracts were screened once duplicates were removed. Then, full texts of 1087 studies were checked and the 156 studies which met eligibility criteria were included for this review (Fig. 1 and Supplementary Appendix S4).

### Description of included studies

All 156 included studies were observational and represented 324,848 obstetric patients admitted to the ICU from 45 different countries (Supplementary Appendix S2). Overall, 26.2% (*n* = 43) reported data from low-income/lower-middle-income countries, 34.5% (*n* = 55) upper-middle-income countries, 40.2% (*n* = 66) high-income countries (Table 1). Four studies were undertaken in more than one country [28–31]. The studies were mainly published in English (83.3% *n* = 130), retrospective (76.3% *n* = 119) and conducted in a single centre (80.1% *n* = 125).

**Table 1:** Studies reporting data on intimate partner violence and social determinants of health among women with severe acute maternal morbidity in the intensive care unit

Descriptors	Low-income/lower-middle income countries^*a*^ (*n* = 43) n (%)	Upper-middle income countries^*a*^ (*n* = 55) n (%)	High-income countries^*a*^ (*n* = 66) n (%)	Total (*n* = 164) n (%)
**Intimate partner violence**	NR	1 (1.8)	NR	1 (0.6)
**Age (any data)**	43 (100.0)	55 (100.0)	66 (100.0)	164 (100.0)
**Marital status**	1 (2.3)	5 (9.1)	5 (7.6)	11 (6.7)
**Place of residence**	6 (14.0)	8 (14.5)	1 (1.5)	15 (9.1)
**Level of education**	9 (20.9)	6 (10.9)	1 (1.5)	16 (9.8)
**Employment rate**	1 (2.3)	2 (3.6)	2 (3.0)	5 (3.0)
**Income**	1 (2.3)	1 (1.8)	NR	2 (1.2)
**Socio-economic status**	4 (9.3)	NR	2 (3.0)	6 (3.7)
**Studies reporting data on race/ethnicity**	NR	7 (12.7)	26 (39.4)^c^	33 (20.1)
**Health insurance** ^*b*^	NR	2 (3.6)	8 (12.1)	10 (6.1)
**Alcohol consumption**	NR	1 (1.8)	2 (3.0)	3 (1.8)
**Smoking**	NR	1 (1.8)	14 (21.2)	15 (9.1)
**Drug use (substance abuse)**	1 (2.3)	2 (3.6)	8 (12.1)	11 (6.7)

*NR* Not reported. See list of references in Supplementary Appendix 4

^*a*^The 156 studies were undertaken in 164 countries

^*b*^Data from one study (Reference 104) were not included only for this item. See list of references in Supplementary Appendix 4

^c^Data were shown from both countries (Australia and New Zealand) in one study (Reference 140 in Supplementary Appendix 4)

### Assessment of study quality

There were 22 (14.1%) studies classified as moderate quality, 95 (60.9%) low quality and 39 (25.0%) very low quality (Supplementary Appendix S3). In some studies, precise definition of the study population was missing with participants referred to as ‘obstetric’ without further definition, or the source of data or case identification method was not stated [32–34].

### Exposure to IPV

Only one study from an upper-middle-income country (Peru) investigated IPV before and during pregnancy among SAMM patients admitted to an ICU, and found that exposure to IPV (aOR 3.83, 95% CI 1.99, 7.37) was significantly associated with SAMM in the ICU (Tables 1 and 2) [35]. Another study undertaken in New Zealand [36] reported that family violence (violence by an intimate partner or other family member) can be a contributory factor for potentially avoidable SAMM in the ICU; and the authors considered that family violence was one of the barriers to access or engage with maternity care. No patients were described as affected by family violence in that study, however no structured process to document family violence was evident. Even though 24 studies reported that trauma was one of the causes of maternal ICU admissions, with rates from 0.5% in Argentina [37] to 14.0% in USA [38], the authors did not report whether IPV was the reason leading to trauma or ICU admission.

**Table 2:** Findings on intimate partner violence and social determinants of health among women with severe acute maternal morbidity in the intensive care unit

Descriptors		Low-income/lower-middle-income countries^*a*^	Upper-middle-income countries^*a*^	High-income countries^*a*^
		Proportion (percentage) of obstetric patients with the descriptor according to each study^*b*^		
**Intimate partner violence**		NR	58.7%^156 ee^	NR
**Age** ^***c***^ **in years, mean (SD)**		26.8 (2.0)	28.4 (2.5)	30.4 (2.1)
**Marital status** **(married** ^***d,e,f***^ **)**		Nigeria 93.1%^**1*d***^	Brazil 10.6%^(136)*v*^, 56.8%^116^, 81.8%^2*e*^ Peru 14.7%^156*ff*^ Venezuela 18.1%^3*f*^	Israel 96.4%^137^ United Kingdom 69.7%^4^ USA 40.7%^5^, 55.1%^(6)^, 60.2%^7^
**Place of residence** **(rural)**		India 54.2%^8^, 55.1%^132^, 68.5%^155^, 84.0%^9^, Nepal 62.5%^120^ Pakistan 73.0%^10^	Brazil 19.3%^127*r*^, 45.7%^116*m*^, 57.7%^136*aa*^ China 33.6%^124*cc*^, 72.3%^123^ Colombia 39.0%^11*cc*^, 42.7%^12^ Venezuela 59.1%^3^	Canada 18.8%^153*bb*^
**Level of education** ^**b**^ **(uneducated or illiterate)**		Egypt 30.1%^113^, 28.3%^**121*g***^ India 39.5%^139*g1*^, 46.0%^9^, 54.2%^8^, 55.8%^13^ Nepal 44.6%^**120*g***^ Nigeria 60.5%^14*g1*^, 80.2%^**1*h***^	Argentina 1.3%^15^ Brazil 3.3%^136*dd*^, 75.3%^116*n*^ China 15.0%^151*z*^ Peru 7.3%^156*gg*^ Venezuela 14.2%^3^	USA 17.9%^147*y*^
**Employment (yes)**		Egypt 6.5%^121^	Brazil 37.9%^116^ Peru 23.9%^156^	United Kingdom 48.5%^4^ USA 48.8%^5^
**Income**	**≤ 5000 Indian Rupees per month**	India 97.0%^9^	Peru 17.4%^156*hh*^	NR
	**< 2000 Indian Rupees per month**	India 74.0%^9^		
**Low socio-economic status**		India 64.1%^155^; 65.6%^16^, 66.9%^139^, 84.7%^17^	NR	Canada 29.3%^153*s*^ United Kingdom 28.0%^131*s*^
**Studies reporting data on race/ethnicity** ^***k***^		NR	Brazil^116, 136^ China^151^ Cuba^18–20^ South Africa^21^	Australia^22*k*, 142^ Australia and New Zealand^140^ France^133*t*^ Hong Kong^23, 24*k*^ Israel^137^ Italy^25*k*^ Netherlands^26, 27^ New Zealand^28, 29^ Saudi Arabia^41^ Singapore^30, 31^ United Kingdom^4, 131^ USA^5–7, 32–35, 117, 147^ (Hawaii^134^)
**Health insurance** ^***i,l***^		NR	Colombia 96.7% social security system^12^ Peru 98.2% Comprehensive National Insurance (SIS)^156*ii*^	USA private: 40.7%^5^ USA government assisted insurance: 24.4%^35^, 37.4%^6*jj*^, 42.4%^32^, 45.0%^117^ 48.7%^147^, 76.8%^34^ (Hawaii 52.6%^134^)
**Alcohol consumption**		NR	Peru 9.2%^156^	Hong Kong 3.0%^24^ USA 6% either alcohol or drugs^35^
**Smoking**		NR	Peru 4.6%^156^	Australia 40.0%^22*j*^ 42.0%^142^ Canada 10.9%^119^ Finland 8.2%^93*w*^ Hong Kong 4.0%^23*l*^, 10.0%^24^ Israel 7.2%^137*x*^ New Zealand 10.0% ^29^, 20.7%^28^ United Kingdom 6.1%^4^, 12.9%^131*s*^ USA 7.3%^147^, 13.1%^7^ (Hawaii 14.1%^134*u*^)
**Drug use** ^**q**^		India 0.0%^39^	Brazil 0.9%^127^ Peru 0.0%^156^	Austria 3.4%^111*p*^ Hong Kong 3.0%^24^, 4.0%^23^ USA^*q*^ 2.2%^6^, 2.9%^39^, 6.0% either alcohol or drugs^35^, 15.0% ^33^, (Hawaii 7.9%^134*u*^)

*NR* Not reported

^*a*^The 156 studies were undertaken from a single to a wide range of settings (43 from low-income/lower-middle-income-countries, 55 upper-middle-income countries and 66 high-income countries). Some studies were undertaken more than once in the same country (Supplementary Appendix 3). See list of references in Supplementary Appendix 4

^*b*^Rates were reported according to each study, otherwise data were presented in percentages rounded to one decimal. Data on educational level from Reference 25 were not shown because authors did not collect this information from all regions

^*c*^Data on age were reported in all studies. Data on age as a mean were meta-analysed using information of studies undertaken in 31 low-income/lower-middle-income-countries, 33 upper-middle-income countries and 44 high-income countries

^*d*^Married/other

^*e*^With fix partner. Data missed in four participants

^*f*^There was 60.8% cohabitant and 21.1% single

^*g*^ Illiterate/read and write for Egypt and less than primary for Nepal

^g1^Uneducated

^*h*^Post-secondary education or less

^*i*^Data from one study were not included (Ref 104) only for this item

^*j*^Missing data in 86 women, making denominator 163 (the total number of maternal ICU admissions were 249)

^*k*^Ethnicity was defined by country of origin

^*l*^2% more were ex-smokers

^*m*^Rate of participants who did not live in the state capital

^*n*^Up to 9 years of study

^*o*^Median, missing data in 11 (23.9%) participants

^*p*^Opioid dependence

^*q*^Number of patients with drug overdose were not reported in one study (Reference 98 in Supplementary Appendix 4)

^*r*^Included inland Amazonas and other states other than the Capital Manau

^*s*^In United Kingdom, data were missed in 105 patients for socioeconomic deprivation (7.4%), 324 patients for BMI (22.9%) and 309 patients for smoking (21.9%)

In Canada, data were shown for the Quintile 1, and data were missed in 14.0%

^*t*^Maternal place of birth was reported

^*u*^Data according to Table 4 of the study

^*v*^Included a total of 13 patients, of which 12 were married and one was with a stable union. In addition, 77.2% (95) for the variable ‘marital status’ and 41.5% (51) for the variable ‘education’ were reported as uninformed by the authors

^*w*^Comprising 2.5% (7) which quit smoking in early stages of pregnancy and 5.7% (16) smoking at any stage of pregnancy

^*x*^Past smoking 5.4% (6) and current smoking 1.8% (2)

^*y*^It was reported 17.9% for less than high school degree

^*z*^This value is for primary school and below

^aa^Rate for cities in other states and other cities in Pernambuco o

^*bb*^ ata were missed in 0.6%

^*cc*^Non-urban residence

^*dd*^There were 41.5% (51) reported as uninformed

^*ee*^This value included both before and during pregnancy

^*ff*^Additionally, 74.3% (81) were cohabitants

^*gg*^This included 7 patients with primary school plus one patient with no formal education

^*hh*^The rate was for ≤ 260.0 US dollars per month. Peruvian monthly minimum wage is 850 Peruvian Soles (approx. 246.5 US Dollars) at the time of the study; five participants did not provide this information. Mean 368.9 ± 159.6 US dollars

^*ii*^There was 0.9% Militar Health Insurance and 0.9% Social National Health Insurance

^*jj*^According to Table 1 of the study

### Social determinants of health

Other than age data regarding social determinants of health were described in very few studies (Tables 1 and 2).

### Age

Age was reported in all studies and women’s mean ages increased gradually from low-income/lower-income to high-income countries (Tables 1 and 2).

### Race, ethnicity and migration status

The role of race, ethnicity, and migration status as independent contributory factors for maternal ICU admission was not clear, and diverse findings were reported in 33 studies (26 from high-income countries and seven upper-middle income countries) (Table 2).

Several studies from USA [39, 40] and UK reported that Black ethnicity [41, 42] was a risk factor for maternal ICU admission; and women with Black ethnicity was associated with an increased risk of ICU death among maternal ICU admissions [39]. Additionally, minority ethnicities - Hispanic women (40), or Asian, Filipino and Native Hawaiian/Other Pacific Islander [43] - were associated with maternal ICU admission in USA and Hawaii, respectively. Madan et al., (2009) [44] reported a high proportion of African American (47.2%) followed by Caucasian women (28.7%) and other races/ethnicities in the ICU, however, the proportion of African American were significantly lower in maternal ICU admissions than obstetric non-ICU admissions (47.2% vs. 53.6%, respectively). On the contrary, Wanderer et al., (2013) [45] reported a higher rate of Black ethnicity in obstetric ICU admissions (47.1%) than obstetric non-ICU admissions (32.5%). Whilst, Small et al., (2012) [46] and Thakur et al., (2016) [38] reported that African American women comprised the majority of maternal ICU admissions in the USA, Sadler et al., (2013) [36] reported no significant differences in admission to the ICU by ethnicity in women with SAMM in New Zealand.

Furthermore, immigration status could be a contributory factor for SAMM in the ICU. Women from sub-Sahara Africa and Eastern Asia experienced increased risk of ICU admission in the Netherlands [47]. The rate of immigrant women in Italy with SAMM in the ICU was higher compared with Italian women (3.0 vs., 1.9 per 1000 deliveries) [48]. In France, obstetric patients born in regions other than Europe or Africa were more likely to be admitted to an ICU with additional severe morbidity criterion (presence of other severe morbidity criteria than ICU admission) [7]; and immigrant population was a risk factor for maternal ICU admission in China [49].

### Marital status

Marital status was described in eleven studies. Five were from high-income countries (Israel [50], UK [41] and USA [39, 44, 46]), five from upper-middle-income countries (Brazil [51–53], Peru [35] and Venezuela [54]) and one from a lower-middle-income country (Nigeria) [55] (Table 2).

The rates of marriage ranged from 10.6% [51] in Brazil to 96.4% in Israel [50]. A higher rate of married women treated in the ICU has been described in some studies, and being married (aOR 3.86, 95% CI 1.27, 11.73) was significantly associated with SAMM in the ICU in a study from Peru [35]. However, no significant difference in the married rate was found between ICU admitted and non-ICU admitted mothers in USA [39, 44] and UK [41]; and between women with SAMM in the ICU and ICU maternal deaths in USA [39] and Nigeria [55].

### Place of residence

Women’s place of residence was reported in fifteen studies, six from low-lower-middle-income countries (India [56–59], Nepal [60] and Pakistan [61]), eight upper-middle-income countries (Brazil [51, 53, 62], China [63, 64], Colombia [65, 66] and Venezuela [54]) and one high-income country (Canada [67]) (Table 2).

A higher rate of maternal ICU admission was reported for women from rural areas. This rate was from 18.8% in Canada [67] to 84.0% in India [59]. Bajwa et al., (2010) [59] found a significant difference between the rate of critically ill obstetric patients from rural areas compared with urban areas; while Gupta et al., (2011) [56] did not find any association of place of residence and distance travelled for reaching the hospital between women with SAMM and maternal mortality cases in an Indian ICU, but the sample size of the latter study was small. In a study from Nepal, all the women who died in the ICU were from rural areas (14.3%), and according to the authors, those deaths may be explained due to late diagnosis and referral, and fewer experienced health personnel in rural areas [60]. On the contrary, urban residence was a factor associated with maternal ICU admission in Canada (OR 1.09, 95% CI 1.02, 1.16) [67].

### Education

Level of education was assessed in sixteen studies, of which nine were from low- lower-middle-income countries (Egypt [68, 69], India [56, 59, 70, 71], Nepal [60] and Nigeria [55, 72], six from upper middle-income countries (Argentina [73], Brazil [51, 53], China [49], Peru [35] and Venezuela [54]) and one study from a high-income country (USA) [40] (Table 2). These studies suggested that a higher proportion of maternity patients admitted to the ICU have lower level of education.

Higher rates of illiteracy among ICU maternity patients were described from 14.2% in Venezuela [54] to 55.8% in India [70], and 60.5% for ‘uneducated’ obstetric patients in Nigeria [72]. In India, Bajwa et al., (2010) [59] reported significantly higher rates of illiteracy (46%) in maternal ICU admissions; however, Gupta et al., (2011) [56] and Igbaruma et al., (2016) [55] did not find significant differences in the maternal education rate between women with SAMM and maternal death cases admitted to an ICU in India and Nigeria, respectively. Poor educational attainment was a risk factor for maternal ICU admission in China [49] and women with higher education were less likely to be admitted to an ICU than obstetric patients without ICU admission, even though this was not shown in the logistic regression analysis in USA [40]. No significant difference was reported between SAMM patients in the ICU and maternity patients not admitted to an ICU in Peru [35].

Rate of illiteracy was significantly higher among maternal death cases than women with SAMM in the ICU in India [70] and Egypt [69]; and a higher rate of maternal mortality was found in uneducated obstetric patients admitted to the ICU in India [71]. Furthermore, Vasquez et al., (2015) [73] found that there was a significantly higher educational level in women with SAMM in the ICU (11.8 ± 4.3 years) than those who died in the ICU (8.3 ± 3 years) in Argentina [73].

### Employment and income

Employment was evaluated in five studies [35, 41, 46, 53, 69] (Table 2), and there were lower rates of employment among obstetric patients admitted to the ICU in studies from lower and upper-middle income countries (Egypt 6.5% [69], Peru 23.9% [35] and Brazil 37.9% [53]) than data found in two studies from high-income countries (USA 48.8% [41] and UK 48.5% [46]). Sultan et al., (2017) [69] reported no significant differences in working status rate between SAMM patients in the ICU and maternal ICU deaths in Egypt; while Ayala Quintanilla et al., (2020) [35] found no difference in the employment rate in SAMM patients in the ICU than parturient not admitted to ICU in Peru.

Income was reported in two studies (India [59] and Peru [35]). Bajwa et al., (2010) [59] indicated that 97.0% of obstetric patients admitted to the ICU had a poor financial status, and a significant number (74.0%) were below the poverty line. In contrast, Ayala Quintanilla et al., (2020) [35] reported that the minority of SAMM patients in the ICU in Peru had a low income (≤ 260.0 US dollars per month).

### Socio-economic status

Socio-economic status was evaluated in six studies, four studies were from a lower-middle-income country (India [57, 71, 74, 75]) and two from high-income countries (UK [42] and Canada [67]), but the results were ambivalent (Table 2). Most obstetric Indian patients admitted to the ICU were from low socioeconomic status with values from 64.1% [57] to 84.7% [75]. Miglani et al., (2020) [71] suggested that maternal outcomes in the ICU were worst in Indian patients with lower socioeconomic status; however, no significant differences in relation to socioeconomic status were found between women with SAMM in the ICU and ICU maternal deaths in another study from India [74]. Residing at the lowest income quintile was a factor associated with maternal ICU admission in Canada (OR 1.44, 95% CI 1.34, 1.55) [67], but no statistically significant differences were observed among quintile groups (socio-economic deprivation) of women admitted to an ICU during pregnancy or postpartum in the UK [42].

### Health insurance

Health insurance was assessed in ten studies. Two studies from upper-middle-income countries described that 96.7% (Colombia) [76] and 98.2% (Peru) [35] of women with SAMM in the ICU had health insurance provided by the government. The other eight studies were from USA [38–40, 45, 46, 77, 78] including Hawaii [43] (Table 2).

In studies from USA, one study showed that majority of pregnant women admitted to ICU (76.8%) had government assisted insurance [38]. In four studies the rates of private insurance ranged from 40.7% [46] to 66.9% [78] and another study described a percentage of 62.6% for commercial insurance or self-pay insurance [39] among maternal ICU admissions. This latter study reported that the type of health insurance was not a risk factor for maternal ICU admission when compared with patients without ICU admission, or between women with SAMM in the ICU and maternal ICU deaths [39]. In contrast, Wanderer et al., (2013) [45] reported a higher usage rate of Medicare/Medicaid (42.4%) in obstetric ICU admissions than obstetric non-ICU admissions (36.8%); Estrada et al., (2021) [43] indicated that pregnant and postpartum women with Medicaid/Medicare had higher rates of ICU admission than those with private health insurance (aOR: 1.69, 95% CI 1.49, 1.96); and Rossi et al., (2019) [40] found that women admitted to the ICU in were more likely to have Medicaid insurance (aOR 1.2, 95% CI 1.2, 1.3) compared with those not admitted to ICU in the peripartum period.

### Substance abuse

#### Alcohol consumption

Two studies from high-income countries reported that the alcohol consumption rate was 3.0% in Hong-Kong [79], and 6.0% for the use of either alcohol or drugs during pregnancy among women admitted to ICU in USA [78] (Table 2). Another study from an upper-middle-income country (Peru) reported no significant differences in alcohol consumption rates between SAMM patients in the ICU (9.2%) and maternity patients not admitted to an ICU (5.5%) [35].

#### Smoking

Data on smoking among maternal ICU admissions were described in fourteen studies from high-income countries (Australia [80, 81], Canada [82], Finland [83], Hong-Kong [79, 84], Israel [50], New Zealand [36, 85], UK [41, 42] and USA [40, 44] including Hawaii [43]) and one upper-middle-income country (Peru) [35], with rates from 4.0% in Hong Kong [84] to 42.0% in Australia [81] (Table 2). Madan et al. (2009) [44] reported that smoking was significantly associated with obstetric ICU admissions; but similar results were not found in the USA [40, 43], UK [42] and Canada [82].

#### Drug use

Eleven studies reported data on drug use from 0.0% in India [30] and Peru [35] to 15.0% in USA [86]. Eight were from high-income countries (Austria [87], Hong-Kong [79, 84] and USA [30, 39, 78, 86], including Hawaii [43], two upper-middle-income countries (Brazil [62] and Peru [35]) and one lower-income country (India [30]) (Table 2). Substance use disorder (aOR 2.10, 95% CI 1.61, 2.74) had an increased likelihood for ICU admission among pregnant and postpartum women in Hawaii [43]. Additionally, drug dependence was significantly associated with maternal ICU admissions and maternal mortality in the ICU in another study undertaken in USA [39].

## Discussion

This systematic review is the first to highlight that while a socio-economic gradient is associated with most rates of illness, few studies described data on social determinants of health other than age in maternal ICU admissions. Furthermore, only one study reported data on exposure to IPV in women with SAMM in the ICU [35].

There was absence of data on IPV among women with SAMM in the ICU. A single study, undertaken in Peru, assessed IPV among women with SAMM in the ICU [35]. This case-control study demonstrated that there was an increased risk of SAMM resulting in ICU admissions for those women exposed to IPV, suggesting a more severe impact of IPV on pregnancy and the mother-baby dyad than has been previously understood. Only one other study [36] hypothesised that family violence could be a contributory factor for SAMM in the ICU; however, as family violence was not routinely screened and recorded in medical reports during pregnancy in New Zealand, the reported absence of family violence may be related to lack of data rather than an absence of violence at the time of the study. This gap in knowledge of IPV exposure among women with SAMM in the ICU is concerning given the nearly four-fold increase reported by Ayala Quintanilla et al., (2020) [35] and the association of IPV with all causes of maternal death [3].

Few studies examined social determinants of health among women with SAMM in the ICU, with just age routinely reported in all studies. Most did not report on socio-economic status, and in those studies that did, there was a higher proportion of women who were poorly educated, from rural areas, and with low incomes, and in high income countries, either Black/Afro-American [7, 40] or others of an immigrant/minority ethnicity background [43, 49]. These indicate that it is important to gain more insight into immigration background, and investigate the relevance of length of migrant status, country of birth or origin, language, and other characteristics that may contribute to access and engagement with a health care service.

Poor socio-economic status is associated with poor health [14], and with IPV [88, 89], consistently across high/middle/low-lower-income countries. The association between maternal mortality, and social and economic factors has been previously reported [90, 91], and a few studies have shown that risk of SAMM may be increased with lower socioeconomic status [92–94].

Social determinants of health, such as poverty level, educational attainment, economic, social and behavioural factors among others, shape the exposure and vulnerability of populations and play an important role in the health of women and their newborns [14]. We suggest that there may be a likely association between socio-economic factors and SAMM in the ICU in the manner previously reported for maternal mortality [90, 91], but there is a lack of data about it. Without including such relevant items in studies, prediction studies have incomplete data when examining factors related to increased likelihood of maternal ICU admission or maternal outcomes, and health service interventions aimed at preventing SAMM will miss targeting the needs of women to be effective. For example, broader interventions, such as improving literacy rates may play a role to reduce SAMM in some regions. Similar to previous systematic reviews, all included studies were observational [10, 11] and described very few data on social determinants of health [11]. We need a better understanding of social determinants of health in women with SAMM to improve maternity outcomes.

Our systematic review has confirmed that there has been no improvement in the consistency of data reported by studies on obstetric ICU patients, and studies are persistently of a low to moderate quality only. Thus, the project to develop a core outcome set for research on critically ill obstetric patients that is underway is timely and should improve data collection in future studies [95].

### Strengths and limitation of this study

Even though one important strength of this review was the effort in the use of the PRISMA Statement [23] and the comprehensive search for relevant studies published in English and Spanish in five databases; we cannot exclude the possibility that there may be other relevant studies published in other languages.

The findings of this review should be seen in light of some limitations. For instance, not all studies reported the primary/secondary outcome investigated in this review, which may influence the present findings. Data on ICU may also be diverse according to criteria used for ICU admission, availability of resources, model of maternal healthcare and healthcare systems across settings and countries. There was high heterogeneity across the studies and different studies applied different definitions for study participants and assessment of social determinants of health. In addition, most studies reported data of the variables of interest of the study only for maternal ICU admissions (which included information from both women with SAMM in the ICU and maternal ICU deaths); and did not show data on the general obstetric population or non- ICU obstetric population; so it was not possible to compare and have a broader understanding of SAMM in the ICU. Furthermore, studies that defined severe maternal morbidity more broadly than ICU admission were excluded in this review and such studies may include relevant data on social health determinants and severe maternal morbidity. Additionally, the quality of the studies ranges from moderate to very low.

## Conclusions

There is a significant evidence gap regarding women with SAMM in the ICU and their social determinants of health, including IPV. These data are essential to better understand the complete picture of the maternal morbidity spectrum and its potential and modifiable causes. A concerted effort should be made to improve data collected on women with SAMM by including social determinants of health and IPV. Our findings suggest that many relevant factors contribute to SAMM and a better understanding of these offer new opportunities to improve maternal health.

### Supplementary Information

**Additional file 1: Supplementary Appendix S1.** General search strategy of studies on women with severe acute maternal morbidity in the intensive care unit.

**Additional file 2: Supplementary Appendix S2.** Characteristics of included studies of women with severe acute maternal morbidity in the intensive care unit.

**Additional file 3: Supplementary Appendix S3.** Quality of the studies on women with severe acute maternal morbidity in the intensive care unit using the Critical Appraisal Skills Programme tool.

**Additional file 4: Supplementary Appendix S4.** List of included studies on women with severe acute maternal morbidity in the intensive care unit.

## Data Availability

The datasets used and/or analysed during the current study are available from the corresponding author on reasonable request. Even though, most data generated or analysed during this study are included in this published article [and its supplementary information files].

## Abbreviations

CASP: Critical Appraisal Skills Programme
ICU: Intensive care unit
IPV: Intimate partner violence
LILACS: Latin American and Caribbean Health Science Information Database
SAMM: Severe acute maternal morbidity
SciELO: Scientific Electronic Library Online
UK: United Kingdom
USA: United States of North America
WHO: World Health Organization
