# Supplementary material for: Social determinants and exposure to intimate partner violence in women with severe acute maternal morbidity in the intensive care unit: a systematic review
Source: BMC Pregnancy Childbirth. 2023 Sep 12;23:656. doi: 10.1186/s12884-023-05927-5 (PMC10496274; doi:10.1186/s12884-023-05927-5)
Supplement: Supplementary file 1 — Additional file 1: Supplementary Appendix S1. General search strategy of studies on women with severe acute maternal morbidity in the intensive care unit. [file 12884_2023_5927_MOESM1_ESM.docx]

**Supplementary Appendix S1**

**General search strategy of studies on women with severe acute maternal morbidity in the intensive care unit*^a^***

| **Search** | **MESH terms and/or free text words** |
| --- | --- |
| #1 | “intensive care unit” OR “intensive care” OR “critical care” OR “critically ill” |
| #2 | “social determinants of health” OR determinants OR social OR socioeconomic OR demographic OR characteristics OR “demographic characteristics” |
| #3 | violence OR “violence against women” OR “gender-based violence” OR “family violence” OR “domestic violence” OR “exposure to violence” OR “battered women” OR “partner abuse” OR “spousal abuse” “spouse abuse” OR “intimate partner violence” |
| #4 | pregnancy OR “pregnancy complications” OR “pregnancy morbidity” OR obstetrics OR mother OR maternity OR “maternal mortality” OR “maternal death” OR “severe maternal morbidity” OR “severe acute maternal morbidity” OR “near miss” OR “severe obstetric morbidity” OR partum OR childbirth OR postpartum OR “postpartum morbidity” OR puerperium OR parturient OR “postnatal morbidity” |
|  |  |
| #5 | #1 AND # 2 AND # 4 Limits: 01/01/2000 to 15/12/2021 in English and Spanish on humans |
| #6 | #1 AND # 3 AND # 4 Limits: 01/01/2000 to 15/12/2021 in English and Spanish on humans |
| #7 | #1 AND # 4 Limits: 01/01/2000 to 15/12/2021 in English and Spanish on humans |

*^a^*This was adapted and modified according to each electronic database.
