# Supplementary material for: Social determinants and exposure to intimate partner violence in women with severe acute maternal morbidity in the intensive care unit: a systematic review
Source: BMC Pregnancy Childbirth. 2023 Sep 12;23:656. doi: 10.1186/s12884-023-05927-5 (PMC10496274; doi:10.1186/s12884-023-05927-5)
Supplement: Supplementary file 2 — Additional file 2: Supplementary Appendix S2. Characteristics of included studies of women with severe acute maternal morbidity in the intensive care unit. [file 12884_2023_5927_MOESM2_ESM.docx]

**Supplementary Appendix S2**

**Characteristics of included studies of women with severe acute maternal morbidity in the intensive care unit**

| **N^o^** | **Author** | **Journal** | **Country** | **Language** | **Study design stated by the authors** | **Temporality indicated by the authors** | **Study period** | **Setting** | **Sample size** |
| --- | --- | --- | --- | --- | --- | --- | --- | --- | --- |
| 1 | Acevedo et al. 2012 | Revista Cubana de Obstetricia y Ginecologia | Cuba | Spanish | Descriptive | Retrospective | Jan 2008 to Dec 2009 | One general teaching hospital with an intensive care unit (ICU). | 212 |
| 2 | Acho et al. 2011 | Revista Peruana de Ginecologia y Obstetricia | Peru | Spanish | Descriptive | Retrospective | Jan 2008 to Dec 2009 | One general tertiary referral teaching hospital. | 52 |
| 3 | Afessa et al. 2001 | Chest | United States of America (USA) | English | - | Retrospective | Jan 1991 to Dec 1998 | One tertiary care university-affiliated with a medical (multidisciplinary) 16-bed ICU. | 74 |
| 4 | Aldawood et al. 2011 | Annals of Saudi Medicine | Saudi Arabia | English | Cohort | Retrospective | Dec 1999 to Dec 2009 | One tertiary care teaching hospital. | 75 |
| 5 | Al-Jabari et al. 2001 | Saudi Medical Journal | Saudi Arabia | English | - | Retrospective | Jun 1994 to Jun 1997 | One university hospital with a surgical ICU. | 63 |
| 6 | Al Suleiman et al. 2006 | Archives of Gynecology and Obstetrics | Saudi Arabia | English | - | Retrospective | May 1992 to April 2004 | One tertiary referral teaching hospital with a 12-bed multidisciplinary ICU. | 64 |
| 7 | Alves et al. 2021 | International Journal of Gynaecology and Obstetrics | Brazil | English | Cohort | Retrospective | Jan 2015 to Dec 2018 | One public, university, tertiary-care maternity hospital with five beds for maternal UCI | 557*^d^* |
| 8 | Anwari et al. 2004 | Saudi Medical Journal | Saudi Arabia | English | - | Retrospective | 1997 to 2002 | One Armed Forces Hospital with a 16-bed medical and surgical ICU. | 99 |
| 9 | Aoyama et al. 2019 | Critical Care | Canada | English | Cohort | Retrospective | 2004 to 2015 | Population study of 342 acute care hospitals | 10,204 |
| 10 | Ashraf et al. 2014 | Anesthesiology Research and Practice | India | English | - | Retrospective | Aug 2012 to Jul 2013 | One tertiary teaching hospital with a 6-bed multidisciplinary ICU. | 55 |
| 11 | Ayala et al. 2020 | Birth | Peru | English | Case-control | Prospective | 2015 to 2016 | One tertiary hospital (national referral hospital for high-risk maternal and neonatal patients) | 109 |
| 12 | Bajwa et al. 2010 | Journal of Emergencies, Trauma and Shock | India | English | - | Retrospective | Dec 2006 to Jan 2010 | One tertiary hospital with a 12 bed-ICU. | 61 |
| 13 | Balestena et al. 2006 | Revista de Ciencias Médicas de Pinar del Rio | Cuba | Spanish | Cross sectional | Retrospective | May 2001 to Dec 2004 | One university hospital with an ICU. | 168 |
| 14 | Baloch et al.  2010 | Journal of Surgery Pakistan | Pakistan | English | Descriptive | Retrospective | Feb 2000 to Jan 2010 | One tertiary teaching hospital with a 7-bed surgical multidisciplinary ICU. | 152 |
| 15 | Bandeira et al. 2014 | International Journal of Gynecology and Obstetrics | Brazil | English | Cohort | Prospective | Jan 2007 to Feb 2009 | One tertiary referral ICU. | 298 |
| 16 | Barry et al. 2018 | Société Francaise d’Anesthé sie et de Réaanimation | France | English | - | Retrospective | Jan 2010 to Dec 2014 | The Medicalisation Programme for Information  Systems for Medicine, Surgery, and Obstetrics (PMSI-MCO) National Hospital Discharge Database of ICUs in French territory. | 16,010 |
| 17 | Bentata et al. 2012 | International Journal of Gynecology and Obstetrics | Morocco | English | - | Retrospective | Jun 2007 to Jun 2011 | One regional hospital with a multipurpose ICU. | 217 |
| 18 | Bhadade et al. 2012 | Indian Journal of Critical Care Medicine | India | English | Cohort | Prospective | Mar 2009 to Aug 2010 | One tertiary teaching hospital with a medical ICU. | 122 |
| 19 | Bibi et al. 2008 | Journal of Ayub Medical College, Abbottabad: JAMC | Pakistan | English | Case Series | Retrospective | Jan to Dec 2006 | One tertiary university hospital with a 16-bed ICU. | 30 |
| 20 | Blanco et al. 2016 | Ghana Medical Journal | Mexico | English | Descriptive | Ambispective | Jun 2009 to Jun 2013 | One maternal-perinatal hospital with an obstetrical ICU. | 232 |
| 21 | Briones et al. 2015 | Revista de la Asociación Mexicana de Medicina Crítica y Terapia Intensiva | Mexico | Spanish | Descriptive | Retrospective | Jun (Jul)^a^ 2014 to Jan 2015 | One general hospital with an Obstetrics and Gynecology ICU. | 180 |
| 22 | Cabezas et al. 2004 | Revista Electrónica de las Ciencias Médicas en Cienfuegos | Cuba | Spanish | Descriptive | Retrospective | Jan 1991 to Dec 2001 | One university hospital with an ICU. | 323 |
| 23 | Chantry et al. 2021 | Anaesthesia Critical Care Pain Medicine | France | English | - | Retrospective | 2010 to 2014 | National study. French national hospital discharge database including all ICUs in France | 15,096 |
| 24 | Chantry et al. 2015 | Critical Care Medicine | France | English | National descriptive | Retrospective | Jan 2006 to Dec 2009 | French national hospital discharge database including all ICUs. | 11,824 |
| 25 | Chawla et al. 2015 | Medical Journal Armed Forces India | India | English | Case Series | Retrospective | Jun 2007 to May 2010 | One tertiary referral teaching hospital of armed forces with an 18-bed multidisciplinary ICU. | 35 |
| 26 | Cheng et al. 2003 | International Journal of Obstetric Anesthesia | Singapore | English | - | Retrospective | Jul 1994 to Jun1999 | One general hospital with a surgical ICU. | 43 |
| 27 | Cohen et al. 2000 | Acta Obstetricia et Gynecologica Scandinavica | Israel | English | Case Series | Retrospective | Jan 1994 to Jul 1998 | One tertiary care university-affiliated hospital with an 8-bed general ICU. | 46 |
| 28 | Crozier et al. 2011 | Australian and New Zealand Journal of Obstetrics and Gynaecology | Australia | English | Audit | Retrospective | Oct 2006 to Sep 2008 | One university-affiliated tertiary referral hospital (Quaternary obstetric unit)  with a 21-bed general ICU. | 60 |
| 29 | Dasgupta et al. 2017 | Indian Journal of Critical Care Medicine | India | English | Cohort | Retrospective | Jan 2011 to Dec 2015 | One general (medical and surgical) critical care unit (CCU) of a government teaching hospital (a tertiary referral centre) | 205 |
| 30 | Dávila Gómez, H. L. et al. | Progresos de Obstetricia y Ginecología | Cuba | Spanish | Descriptive | Retrospective | Jan 2002 to Dec 2010 | One general teaching hospital with an ICU. | 42 |
| 31 | De Greve et al. 2016 | Gynecologic and Obstetric Investigation | Belgium | English | - | Retrospective | Jan 2000 to July 2012 | One tertiary care hospital with a medical, surgical and cardiac ICUs. | 183 (with 190 admissions) |
| 32 | Demirkiran et al. 2003 | International Journal of Obstetric Anesthesia | Turkey | English | - | Retrospective | Jun 1995 to Jun 2000 | One multidisciplinary ICU. | 125 |
| 33 | Diaz et al. 2006 | Revista Cubana de Obstetricia y Ginecología | Cuba | Spanish | Descriptive | Retrospective | Jan 1999 to Dec 2004 | One general teaching hospital with an ICU. | 291 |
| 34 | Donati et al. 2012 | Acta Obstetricia et Gynecologica Scandinavica | Italy | English | - | Retrospective | 2004 to 2005 | ICUs or coronary care units from six Italian regions [Piedmont and Emilia Romagna (northern), Tuscany and Lazio (central) and Campania and Sicily (southern)]. | 1,259 |
| 35 | Estrada Altamirano, A. et al. 2002 | Perinatologia y Reproduccion Humana | Mexico | Spanish | - | Retrospective | Jan 1993 to Dec1998 | One national perinatal institute with an obstetric ICU. | 1,913^b^ |
| 36 | Estrada et al. 2021 | Public health reports | Hawai (USA) | English | Cohort | Retrospective | Jan 2012 to Sep 2017 | Hawaii Health Information Corporation (HHIC) inpatient database (from all hospitals in Hawaii) | 1,340 |
| 37 | Fadiloglu et al. 2019 | Journal of Perinatal Medicine | Turkey | English | - | Retrospective | 2007 to 2017 | One tertiary university hospital | 160 |
| 38 | Farr et al. 2017 | Wien Klin Wochenschr | Austria | English | - | Retrospective | Jan 2011 to Dec 2014 (for the study group), and Jan 1996 to Dec 2003 (for the historical group). | One ICU at the Medical University of Vienna, Vienna General Hospital (a tertiary referral center) | 238 |
| 39 | Farzi et al. 2017 | Anesthesiology and Pain Medicine | Iran | English | Cohort | Retrospective | Apr 2009 to Apr 2014 | One ICU of Alzzahra teaching hospital affiliated to Guilan University of Medical Sciences | 1,019 |
| 40 | Fong et al. 2020 | Revista Información Científica | Cuba | Spanish | Descriptive, cross-sectional | - | Jan 2014 to Dec 2019 | One general teaching hospital | 223 |
| 41 | Fouly et al. 2018 | Applied Nursing Research | Egypt | English | Cross sectional | Prospective | Mar to Aug 2015. | ICU belong to maternity services with 12 beds and it was also possible to use the general ICU at the Assiut Main University Hospital; and, a general ICU at the Alfayoum University Hospital. | 93 |
| 42 | Franco-Sansaloni et al. 2017 | Ginecología y Obstetricia de México | Spain | Spanish | Descriptive observational | Retrospective | Jan  2010 to Jun 2015. | One ICU of a Hospital. | 34 |
| 43 | Galvez et al. 2009 | Revista Colombiana de Obstetricia y Ginecologia | Colombia | Spanish | - | Retrospective | Jul 2004 to Feb 2007 | One tertiary teaching hospital with a 9-bed ICU. | 46 |
| 44 | Garcia et al. 2009 | Anestesia en México. | Mexico | Spanish | Cross sectional | Retrospective | Jan 2007 to Dec2008 | One general hospital with an ICU. | 50 |
| 45 | Ghike et al. 2012 | Journal of South Asian Federation of Obstetrics and Gynaecology | India | English | - | Retrospective | Nov 2009 to Oct 2011 | One hospital with an ICU. | 47 |
| 46 | Gilbert et al. 2003 | Obstetrics & Gynecology | USA | English | - | Retrospective | Jan 1991 to Dec 1998. | One tertiary care university-affiliated hospital (perinatal referral center) with a 30-bed ICU that contains.  separate medical, surgical, and coronary care units | 233 |
| 47 | Godeberge et al. 2021 | Anesthesia & Analgesia | France | English | - | Retrospective. Secondary analysis of the epidemiology of severe acute maternal morbidity (EPIMOMS) study, a prospective population-based study specifically designed to study SAMM in 6 French regions. | 2012 to 2013 | Population-based study (119 maternity units and 136 ICUs) | 511 |
| 48 | Gombar et al. 2014 | Journal of Anaesthesiology Clinical Pharmacology | India | English | - | Retrospective | Jun 2007 to May 2012 | One tertiary hospital with a 14-bed multidisciplinary ICU. | 164 (151 for analysis) |
| 49 | Gonzales et al. 2015 | Revista Cubana de Obstetricia y Ginecologia | Cuba | Spanish | Cross sectional | - | Jan 2010 to Dec 2014 | One university general hospital with an ICU. | 504 |
| 50 | Gupta et al. 2021 | Indian Journal of Critical Care Medicine | India | English | - | Retrospective | Oct 2018 to Mar 2020 | One tertiary institute (Government Medical College, Jammu) | 127 |
| 51 | Gupta et al. 2011 | Indian Journal of Anaesthesia | India | English | - | Prospective | Apr 2009 to Mar 2010 | One tertiary hospital with a 3-bed obstetric ICU. | 24 |
| 52 | Harrison et al. 2005 | Critical Care | United Kingdom (UK) | English | - | Retrospective | Dec 1995 to Jun 2003 | 159 critical care unit s from the Case Mix Program database (national comparative  audit of adult, general critical care units) coordinated by the Intensive Care National Audit and Research Centre. | 1,902 |
| 53 | Hasbun et al. 2013 | Revista Medica de Chile | Chile | Spanish | Case Series | Retrospective | Jan 2006 to Dec 2010 | One tertiary referral teaching hospital. | 89 |
| 54 | Hazelgrove et al. 2011 | Critical Care Medicine | UK | English | - | Retrospective | Jan 1994 to Dec 1996 | Fourteen ICUs (by using the South-West Thames Database). | 210 |
| 55 | Hernandez et al. 2020 | Medisur | Cuba | Spanish | Descriptive, cross-sectional, case series | - | Jan 2016 to Dec 2018 | One hospital | 145 |
| 56 | Igbaruma et al. 2016 | International Journal of Obstetric Anesthesia | Nigeria | English | Observational | Prospective | Jan 2009 to Dec 2012 | One referral teaching hospital. | 101 |
| 57 | Jain et al. 2016 | International Journal of Gynecology and Obstetrics | India | English | - | Prospective | Oct 2010 to Dec 2011 | One government tertiary care teaching hospital. | 90 |
| 58 | Jardine et al. 2021 | BJOG: An International Journal of Obstetrics & Gynaecology | UK | English | Cohort | Retrospective | Apr 2015 to Mar 2016 | National maternity data from maternity and intensive care units in England and Wales | 1414 |
| 59 | Jayaratnam et al. 2020 | The Australian & New Zealand Journal of Obstetrics & Gynaecology | Australia | English | Observational | Retrospective | Jan 2013 to Dec 2017 | One hospital with 10 ICU beds | 69 |
| 60 | Joseph et al. 2018 | Indian Journal of Anaesthesia | India | English | - | Ambispective | Oct 2015 to Sep 2016 and from Oct 2010 to Sep 2015. | One Multidisciplinary ICU of a tertiary care hospital. | 109 |
| 61 | Kallur et al. 2014 | Journal of Clinical and Diagnostic Research | India | English | Not indicated | Retrospective | Jan 2011 to Dec 2012 | One maternal and new-born tertiary hospital with a maternal ICU. | 85 |
| 62 | Karnad et al. 2004 | Critical Care Medicine | India | English | - | Retrospective | Jan 1997 to Dec 2001 | One large tertiary referral care center and university hospital with 17-medical-neuro (multidisciplinary) ICU which also received obstetric admission from a near Maternity hospital. | 453 |
| 63 | Karolinski et al 2010 | International Journal of Gynecology and Obstetrics | Argentina, Uruguay | English | Descriptive (nested in a cohort study) | Prospective | Jan to Dec 2005 | 24 public hospitals (20 in Argentina and 4 in Uruguay). | 80 |
| 64 | Keizer et al. 2006 | European Journal of Obstetrics & Gynecology and Reproductive Biology | Netherlands | English | - | Retrospective | 1990 to 2001 | One tertiary care university center with a 30-bed ICU. | 142 |
| 65 | Khergade et al. 2020 | Indian Journal of Critical Care Medicine | India | English | Cohort | Prospective | Not indicated | One tertiary care referral teaching institute with 15-bedded Obstetric Critical Care Unit. | 250 |
| 66 | Krawczyk et al. 2021 | Ginekologia Polska | Poland | English | - | Retrospective | Jan 2007 to Dec 2014 | One obstetric tertiary care center with a four-bed ICU | 266 |
| 67 | Kumar et al. 2021 | The Journal of Obstetrics and Gynecology of India | India | English | Cohort | Retrospective | Jan 2019 to Dec 2020 | One tertiary care hospital with a 15-bedded ICU | 22 |
| 68 | Lapinsky et al. 2011 | Journal of Critical Care | Australia, Belgium, USA, Canada, South Africa | English | - | Retrospective | 1994 to 1998 | Five hospitals with ICUs. | 332 |
| 69 | Lataifeh et al 2010 | Journal of Obstetrics and Gynaecology | Jordan | English | - | Retrospective | Jan 2002 to Dec 2008 | One tertiary university hospital with a 12-bed multidisciplinary ICU | 43 |
| 70 | Lawton et al. 2010 | Australian and New Zealand Journal of Obstetrics and Gynaecology | New Zealand | English | Audit | Retrospective | 2005 to 2007 | One tertiary hospital with an ICU. | 29 |
| 71 | Leung et al. 2010 | Hong Kong Medical Journal | Hong Kong | English | Cohort | Retrospective | Jan 1998 to Dec 2007 | One regional hospital with a 20-bed ICU | 50 |
| 72 | Lin et al. 2019 | Medicine | China | English | - | Retrospective | Jan 2009 to Dec 2016 | One obstetric center at Third Affiliated Hospital of Guangzhou Medical University | 426 |
| 73 | Lotufo et al. 2012 | Clinics | Brazil | English | Cross sectional | Retrospective | Jan2004-Dec2007 (4y) | One teaching general hospital (referral center) with and 18-bed adult ICU | 158 |
| 74 | Loverro et al. 2001 | Archives of Gynecology and Obstetrics | Italy | English | - | Retrospective | 1987 to 1998 | One department of Obstetrics and Gynecology, University of Bari. | 41 |
| 75 | Madan et al 2009 | Journal of Maternal-Fetal & Neonatal Medicine | USA | English | - | Retrospective | Jan 1997 to Dec 2005 | A perinatal linked dataset provided by the Maternal  Child Health Epidemiology in New Jersey. | 15,447 |
| 76 | Maiden et al. 2020 | BJOG: an International Journal of Obstetrics and Gynaecology | Australia and New Zealand | English | Cohort | Retrospective | Jan 2008 to Dec 2017 | Multicentric and multicountry study. Data from the Australia and New Zealand Intensive Care Society (ANZICS) Adult Patient Database and national agencies, which included 183 ICUs in Australia and New Zealand. | 16,063 (from Australia and New Zealand) |
| 77 | Malpica et al. 2009 | Revista Medica Electronica | Cuba | Spanish | - | - | Jan 2003 to Dec 2006 | One general teaching hospital with an ICU. | 83 |
| 78 | Malvino et al. 2014 | Revista Argentina de Terapia Intensiva | Argentina | Spanish | Case series, descriptive and analytic | - | Mar 1991 to Jul 2013 | One private hospital. | 1,005 |
| 79 | Miglani et al. 2020 | Indian Journal of Critical Care Medicine | India | English | Observational | Prospective | Jul 2017 to Dec 2018 | One hospital with 10 bedded ICU | 124 |
| 80 | Mirghani et al. 2004 | International Journal of Obstetric Anesthesia | United Arab Emirates | English | Audit | Retrospective | Jan 1997 to Dec 2002 | One tertiary referral teaching hospital with an 8-bed ICU. | 60 |
| 81 | Mjahed et al. 2006 | Journal of Obstetrics and Gynaecology | Morocco | English | - | Retrospective | Jan 1995 to Dec 2002 | One university teaching hospital with a 12-bed multidisciplinary surgical ICU. | 364 |
| 82 | Montes et al. 2018 | Revista Da Escola de Enfermagem | Brazil | English | Cross-sectional | Retrospective | Jan 2010 to Dec 2014 | One maternal ICU of the Maternity School of a tertiary referral maternity from a University | 560 |
| 83 | Montoya et al. 2011 | Revista Cubana de Medicina Militar | Cuba | Spanish | Cross sectional and descriptive | - | Jan 2006 to Jun 2008 | One military hospital with an adult ICU. | 26 |
| 84 | Muench et al. 2008 | Journal of Reproductive Medicine | USA | English | - | Prospective | 24-month study period | One tertiary referral university hospital with an ICU. | 34 |
| 85 | Munnur et al. 2005 | Intensive Care Medicine | USA and India | English | - | Retrospective | 1992 to 2001 | One referral hospital for high-risk obstetrics with one-bed obstetric ICU and one 16-bed medical ICU in USA, and one tertiary referral hospital with a 17-bed multidisciplinary ICU in India. | 174 in USA and 754 in India |
| 86 | Murphy et al. 2002 | European Journal of Obstetrics & Gynecology and Reproductive Biology | UK | English | Cohort | Retrospective | 1988 to 1999 | One university teaching hospital | 50 |
| 87 | Nava et al. 2016 | Revista Chilena de Obstetricia y Ginecología | Venezuela | Spanish | Descriptive | Retrospective | 2011 to 2014 | One maternity referral hospital. | 232 (out of 548 which was the total population in the study period) |
| 88 | Ng et al. 2014 | Hong Kong Medical Journal | Hong Kong | English | Case Series | Retrospective | Jan 2006 to Dec 2010 | One regional hospital with a 14-bed ICU | 67 |
| 89 | Ngene et al. 2013 | South African Medical Journal | South Africa | English | Cohort | Prospective | Jul 2010 to Apr 2011 | One regional hospital with a 6-bed mixed medical-surgical ICU; and,  one tertiary hospital with a 5-bed mixed  medical-surgical  ICU and a 4-bed coronary care unit. | 84 |
| 90 | Okafor et al. 2014 | International Journal of Obstetric Anesthesia | Nigeria | English | - | Retrospective | Jan 1997 to Dec 2002 | One tertiary teaching hospital. | 18 |
| 91 | Oliveira et al. 2019 | Critical Care Medicine | Brazil | English | Cohort | Retrospective | Jan 2013 to Dec 2015 | One tertiary public teaching hospital witn an obstetric ICU | 279 |
| 92 | Oliveira, S. et al. 2019 | Acta Médica Portuguesa | Portugal | English | - | Retrospective | Jan 2000 to Dec 2017 | One tertiary care centre in Portugal with a 29-bed ICU | 93 |
| 93 | Oliveira et al. 2009 | International Journal of Gynecology and Obstetrics | Brazil | English | Cohort | Retrospective | Aug 2002 to Sep 2007 | One tertiary university teaching - referral maternity hospital - with a 6-bed general ICU. | 673 |
| 94 | Orsini et al. 2011 | Journal of Clinical Medicine Research | USA | English | - | Prospective | Jun 2009 to Jun 2012 | One general, community inner-city hospital with and 12-bed ICU. | 19 |
| 95 | Osinaike et al. 2006 | The Internet Journal of Anesthesiology | Nigeria | English | Observational | Retrospective | Jan 1997 to Dec 2002 | One tertiary university teaching hospital with a 7-bed general ICU. | 70 |
| 96 | Oud 2017 | Journal of Clinical Medicine Research | USA | English | Population-based cohort | Retrospective | 2001 to 2010 | The Texas Inpatient  Public Use Data File with data of all hospital discharges in the state. | 158,410 |
| 97 | Ozumba et al. 2018 | Indian Journal of Critical Care Medicine | Nigeria | English | - | Retrospective | Jan 2012 to Dec 2013 | One ICU at the Federal Teaching Tertiary Hospital | 89 |
| 98 | Panchal et al. 2000 | Anesthesiology | USA | English | Case- control | Retrospective | Jan 1984 to Dec 1997 | The Uniform Health Discharge Data Set (UHDDS) from Maryland included data of 37 hospitals | 1,023 |
| 99 | Panda et al. 2018 | Indian Journal of Obstetrics and Gynecology of India | India | English | Not indicated | Retrospective | Jul 2015 to Jun 2017 | One tertiary care center (referrals hospital) with a 16-bedded ICU | 92 |
| 100 | Paternina et al. 2015 | Journal of Intensive Care Medicine | Colombia | English | Cohort | Retrospective | Jan 2006 to Dec 2011 | One maternity hospital (public teaching hospital) with a 10-bed medical and surgical ICU. | 726 |
| 101 | Paumier et al. 2020 | Revista Informacion Cientifica | Cuba | Spanish | Cross-sectional | Prospective | Jan to Dec 2019 | One teaching hospital with an ICU | 66 |
| 102 | Paxton et al. 2014 | Australian and New Zealand Journal of Obstetrics and Gynaecology | Australia | English | Observational | Retrospective | Jan 2007 to Jun 2009 | One large metropolitan (tertiary) hospital co-located  with a quaternary-level maternity teaching hospital (referral center) with a 11-bed ICU. | 249 |
| 103 | Perez et al. 2008 | MEDICC Review | Cuba | English | Cohort | Prospective | Jan 1998 to Dec 2004 | One general teaching hospital with a multipurpose 17-bed ICU. | 312 |
| 104 | Porreco et al. 2010 | Journal of Maternal-Fetal and Neonatal Medicine | USA | English | Not indicated | Retrospective | 2004 to 2008 | One hospital with a 23-bed ICU including a 10-bed Pediatric ICU. | 45 |
| 105 | Prats et al. 2011 | Revista de Ciencias Medicas de Pinar del Rio | Cuba | Spanish | Descriptive and longitudinal | Retrospective | Jan 2000 to Dec 2010 | One general university hospital with an ICU. | 561 |
| 106 | Prin et al. 2019 | International Journal of Obstetric Anesthesia | Malawi | English | Cohort | Prospective | Sep 2016 to Mar 2018 | One ICU of Kamuzu Central Hospital | 98*^e^* |
| 107 | Quah et al. 2001 | Annals of the Academy of Medicine, Singapore | Singapore | English | - | Retrospective | Jan 1998 to Dec 1999 | One tertiary referral hospital for obstetrics, gynecology and pediatric patients with a 5- bed adult ICU. | 239 (232)^c^ |
| 108 | Qureshi et al. 2016 | Journal of the Royal Society of Medicine Open | Pakistan | English | - | Retrospective | Jan 2005 to Dec 2014 | One university hospital -tertiary and referral center- with a 10-bed ICU. | 194 |
| 109 | Raad et al. 2003 | Revista electrónica Dr. Zoilo E. Marinello Vidaurreta | Cuba | Spanish | - | Retrospective | Jan 1998 to Dec 1999 | One general teaching hospital with an ICU | 204 |
| 110 | Ramachandra et al. 2013 | Indian Journal of Critical Care Medicine | India | English | - | Retrospective | Jan 2005 to Jun 2011 | One teaching general hospital (tertiary referral center) with a 20-bed medical ICU and 7-bed cardiac ICU. | 65 |
| 111 | Ramlakhan et al. 2021 | BMC Womens's Health | Netherlands | English | Cohort | Retrospective | Jan 2000 to Jan 2016 | One regional tertiary referral center (Level 3 ICU) | 265 |
| 112 | Rathod et al. 2016 | The Journal of Obstetrics and Gynecology of India | India | English | - | Retrospective | Jul 2010 to Jun 2013 | One hospital attached to a medical college with a 6-bed obstetric ICU. | 765 |
| 113 | Richa et al. 2008 | Lebanese Medical Journal | Lebanon | English | - | Retrospective | Jan 1998 to Dec 2005 | One tertiary level III and perinatal center -university affiliated hospital with a 28-bed surgical ICU (the study was undertaken in an 8-bed surgical ICU). | 15 |
| 114 | Rios et al. 2012 | International Journal of Gynecology and Obstetrics | Argentina | English | Descriptive and cohort | Retrospective | Dec 2008 to Dec 2010 | Four medical -surgical ICUs of 4 hospitals. | 242 |
| 115 | Rojas et al. 2011 | Clinica e Investigacion en Ginecologia y Obstetrica | Colombia | Spanish | Descriptive | Retrospective | Aug 2005 to Dec 2007 | One maternity referral hospital with an ICU. | 214 |
| 116 | Rojas JA, Cogollo M, Miranda JE, et al. 2011 | Revista Colombiana de Obstetricia y Ginecologia | Colombia | Spanish | Cohort | Retrospective | Jan 2006 to Dec 2008 | One maternity referral hospital with an ICU. | 346 |
| 117 | Rossi et al 2019 | Obstetrics & Gynecology | USA | English | Population-based cohort | Retrospective | 2012 to 2016 | Population study | 27,602 |
| 118 | Rottenstreich et al. 2020 | Israel Medical Association Journal: IMAJ | Israel | English | Cohort | Retrospective | Jan 2005 to Jul 2013 | One obstetric referral center | 111*^f^* |
| 119 | Rudakemwa et al. 2021 | BMC Pregnancy and Childbirth | Rwanda | English | Cross-sectional | Prospective | Mar 2017 to Feb 2018 | Two public tertiary hospital with six and seven beds for ICU | 94 |
| 120 | Ryan et al. 2017 | Journal of Obstetrics & Gynaecology Canada | Canada | English | Case-control | Retrospective | Jan 2000 to Dec 2011 | Two tertiary obstetric units. | 46 |
| 121 | Sadler et al. 2013 | American Journal of Obstetrics and Gynecology | New Zealand | English | - | Retrospective | 2010 to 2011 | One tertiary teaching hospital (city hospital with tertiary level maternity and neonatal units) with an ICU. | 42 |
| 122 | Saif et al. 2013 | Anaesthesia, Pain & Intensive Care | India | English | Cohort | Prospective | Sep 2009 to Aug 2011 | One tertiary referral teaching hospital with a 5-bed ICU. | 283 (224 for analysis) |
| 123 | Sailaja et al. 2019 | Indian Journal of Critical Care Medicine | India | English | Observational | Prospective | 2-year period | One tertiary care teaching hospital with a multidisciplinary ICU | 91 |
| 124 | Scarlett et al. 2009 | International Journal of Gynecology and Obstetrics | Jamaica | English | - | Retrospective | Jan 2001 to Dec 2006 | One tertiary referral university hospital with a two 8-bed ICUs. | 57 |
| 125 | Selo-Ojeme et al. 2005 | Archives of Gynecology and Obstetrics | UK | English | Case-control | Retrospective | Jan 1993 to Dec 2003 | One tertiary hospital with an ICU. | 33 |
| 126 | Seppanen et al. 2016 and Seppanen 2020 | Intensive and Critical Care Nursing | Finland | English | - | Retrospective audit | 2007 to 2011 | Databases (the APACHE III database, hospital’s database and Medical Birth Register).  Four university hospitals. | 291 (283 with parturient and infant data published in 2020) |
| 127 | Shaikh et al. 2013 | Medical Channel | Pakistan | English | Descriptive | Prospective | Jul 2011 to Jun 2012 | One university hospital with a general ICU. | 83 |
| 128 | Sheela et al. 2004 | Journal of Obstetrics and Gynecology of India | India | English | - | Retrospective | Jan 2000 to Dec 2002 | One tertiary referral hospital (various ICUs). | 108 |
| 129 | Shresta et al. 2017 | Kathmandu University Medical Journal | Nepal | English | - | Prospective | Jan2010 to Dec 2015 | One ICU at the Dhulikhel Hospital, Kathmandu University Hospital (referral hospital). | 56 |
| 130 | Silva | Revista Baiana de Enfermagem | Brazil | English | Quantitative descriptive | Retrospective | Jan 2017 to Dic 2018 | One Intensive Care Unit of a public hospital in Pernambuco | 123*^g^* |
| 131 | Simsek et al. 2011 | Turkish Journal of Medical Sciences | Turkey | English | - | Retrospective | Jan 1999 to Apr 2009 | One ICU | 63 |
| 132 | Simpson et al. 2020 | Critical Care Medicine | UK | English | Cohort study | Data were collected prospectively from 236 adult general ICUs Case Mix Programme (CMP) Database. | Jan 2007 to Dic 2016 | National Database (Data from the Intensive Care National Audit and Research Centre (ICNARC) CMP, which is the national clinical audit for 236 adult critical care in England, Wales, and Northern Ireland). | 15,480 |
| 133 | Small et al. 2012 | Obstetrics & Gynecology | USA | English | - | Prospective | Jan 2005 to Apr 2011 | One tertiary teaching hospital (referral center for women with congenital heart disease) with surgical, medical, cardiac, pulmonary, and neurology ICUs. | 94  (86 for analysis) |
| 134 | Sriram et al. 2008 | Critical care and resuscitation: journal of the Australasian Academy of Critical Care Medicine | Australia | English | Audit | Retrospective | Jan 1998 to Jun 2006 | One tertiary, university-affiliated teaching  hospital with a 24-bed general medical  and surgical ICU. | 56 |
| 135 | Stevens et al. 2006 | American Journal of Obstetrics and Gynecology | USA | English | - | Retrospective | Jan 2002 to May 2004 | One tertiary hospital with a medical ICU, a surgical ICU, a cardiac ICU and a neurological ICU. | 58 |
| 136 | Sultan et al. 2017 | Eastern Mediterranean Health Journal | Egypt | English | - | Prospective | Jan to 31 Dec 2014 | One maternal ICU of El Shatby University Hospital | 448 |
| 137 | Taylor et al. 2000 | South African Medical Journal | South Africa | English | - | Retrospective | Jan 1985 to Dec 1996 | One hospital with a multidisciplinary ICU | 61 |
| 138 | Tempe et al. 2007 | Indian Journal of Medical Sciences | India | English | - | Retrospective | May 2002 to Oct 2004 | One tertiary university teaching hospital with a 7-bed multidisciplinary medical ICU. | 57 |
| 139 | Thakur et al 2016 | Maternal & Child Health Journal | USA | English | - | Retrospective | January 2006 to December 2010 | Five medical center hospitals with a medical  ICU, a surgical ICU, a cardiac ICU, a neurology critical care  unit and the burn center. | 69 |
| 140 | Thakur et al 2015 | Journal of Nepal Health Research Council | Nepal | English | - | Prospective | Jan to Dec 2012 | One tertiary referral hospital with a maternal ICU. | 192 |
| 141 | Togal et al. 2010 | Journal of Critical Care | Turkey | English | - | Retrospective | Jan 2006 to Jul 2009 | One tertiary referral hospital with a 20-bed ICU. | 73 |
| 142 | Tripathi et al. 200 | International Journal of Gynecology and Obstetrics | India | English | Case record | Retrospective | Sep 1993 to Aug 1998 | One centre (Maulana Azad  Medical College and associated Lok Naya113Hospital). | 50 |
| 143 | Urbay et al 2002 | Medicentro | Cuba | Spanish | - | Retrospective | Jan 1992 to Oct 2000 | One university hospital with an ICU. | 275 |
| 144 | Vasquez et al. 2015 | Critical Care Medicine | Argentina | English | National cohort | Prospective | Jan to Dec 2012 | Multicenter, prospective, national cohort study (twenty ICUs, 8 publics and 12 privates -95% of them were referral and medical-surgical ICU). | 362 |
| 145 | Vasquez et al. 2014 | Journal of Critical Care | Argentina | English | Cohort | Prospective | Jan 2008 to Sep 2011 | One private referral clinic with a 12-bed ICU and one university public referral hospital with 14-bed ICU. | 151 |
| 146 | Vasquez et al. 2007 | Chest | Argentina | English | Cohort | Retrospective | Jan 1998 to Sep 2005 | One university-affiliate public hospital with an 8-bed medical and surgical ICU. | 161 |
| 147 | Vargas et al. 2019 | Indian Journal of Critical Care Medicine | Italy | English | Cohort | Retrospective | Jan 2008 to Dec 2013 | One university hospital with 14 beds in a general ICU | 66 |
| 148 | Vieira et al. 2016 | Revista Brasileira de Terapia Intensiva | Brazil | English | Cross-sectional | - | Jan 2012 to Dec 2014 | One maternity hospital, reference center, with a 4-bed maternal ICU. | 373 |
| 149 | Wanderer et al. 2013 | Critical Care Medicine | USA | English | Descriptive | Retrospective | 1999 to 2008 | Maryland State Inpatient Database | 2,927 |
| 150 | Yi et al. 2018 | Obstetrics & Gynecology Science | Korea | English | Observational | Retrospective | Sept 1994 to Oct 2015 | One ICU at the tertiary referral hospital | 176 |
| 151 | Yousuf et al. 2015 | Journal of the Pakistan Medical Association | Pakistan | English | Observational case series | Retrospective | Aug 2011 to Jun 2013 | Department of Obstetrics and Gynecology, Medical University. | 150 |
| 152 | Yuel at al. 2008 | Journal of Obstetrics and Gynecology of India | India | English | Not indicated | Retrospective | Jul 2002 to Jun 2005 | One general hospital with an ICU. | 55 |
| 153 | Yuqui et al. 2017 | Online Clinical Investigations | China | English | Analytical, descriptive, observational. | Retrospective | Jan 2009 to Dec 2016. | One ICU of The Second Clinical Hospital of Fujian Medical University of China | 487 |
| 154 | Zhao et al. 2018 | Critical Care Medicine | China | English | Multicentre cohort | Retrospective | Jan 2008 to Dec 2016 | Three ICUs in tertiary hospitals | 491 |
| 155 | Zorrilla el at. 2017 | Revista Nacional (Itaugua) | Paraguay | Spanish | Descriptive, observational, cross sectional. | Retrospective | Jan to Oct 2015 | One ICU from a national hospital | 135 |
| 156 | Zwart et al. 2010 | Intensive Care Medicine | Netherlands | English | Population (national) based-cohort | Prospective | Aug 2004 to Aug 2006 | 98 Dutch maternity units (10 tertiary care  centers, 33 non-academic teaching hospitals and 55 general hospitals). | 847 |

*^a^*The authors indicated that the study period was from 1 June 2014 to 1 January 2015 in the introduction, but it was stated from 1 July 2014 to 1 January 2015 (results part, first paragraph).

*^b^*It was calculated considering the information in the table 1 of this study.

*^c^*Of 239 obstetric patients, 7 women were re-admitted to the ICU.

*^d^*557 were included from a total of 610 eligible ICU admissions.

*^e^*The total number of maternal ICU admissions were 105.

*^f^*The total number of maternal ICU admissions were 112.

*^g^* Out of 205 medical records, there was a random draw of 123 records, which were included in the study.
