# Supplementary material for: Social determinants and exposure to intimate partner violence in women with severe acute maternal morbidity in the intensive care unit: a systematic review
Source: BMC Pregnancy Childbirth. 2023 Sep 12;23:656. doi: 10.1186/s12884-023-05927-5 (PMC10496274; doi:10.1186/s12884-023-05927-5)
Supplement: Supplementary file 3 — Additional file 3: Supplementary Appendix S3. Quality of the studies on women with severe acute maternal morbidity in the intensive care unit using the Critical Appraisal Skills Programme tool. [file 12884_2023_5927_MOESM3_ESM.docx]

**Supplementary Appendix S3**

**Quality of the studies on women with severe acute maternal morbidity in the intensive care unit using the Critical Appraisal Skills Programme tool**

| **CASP** | **Very Low** | **Low** | **Moderate** |
| --- | --- | --- | --- |
|  | **(n=39 studies)** | **(n=95)** | **(n=22 studies)** |
|  | **Overview comments** | | |
| **1. Did the study address a clearly focused issue?** | Yes, in most studies. | Yes, in all studies. | Yes, in all studies. |
| **2. Was the Cohort /Cases-Controls? Sample of participants recruited in an acceptable way?** | Yes, in more than half studies. | Yes, in more than half studies. | Yes, in most studies. |
| **3. Was severe maternal morbidity accurately measured to minimize bias?** | Yes, in some studies. | Yes, in more than half studies. | Yes, in most studies. |
| **4. Were other characteristics/ outcomes accurately measured to minimize bias?** | Data were mainly retrospectively collected (medical records or other sources).  In some studies, no explanation about which data were collected or from where sources. | In some studies, no explanation about which data were collected or from where sources. | Yes, in most studies. |
| **5. a) Have the authors identified all important cofounding factors? 5. b) Have they taken account of the confounding factors in the design and/or in their analysis?** | Age was identified among all social determinants in most studies.  Other social determinants were assessed in few studies.  IPV was not identified in all studies. | Age was identified among all social determinants in most studies.  Other social determinants were assessed in some studies.  IPV was not identified in all studies. | Age and other social determinants were identified in half studies.  IPV was not identified in all studies. |
| **6. a) Was the follow up of the subject complete enough? 6. b) Was the follow up of the subject long enough?** | Most studies were retrospective.  No information in 3 prospective studies. | Most studies were retrospective.  No information in some cohort or prospective studies. | Most studies were retrospective.  No information in one prospective cohort study. |
| **7. What are the results of this study?** | Some mistakes presenting findings in some studies. | Some mistakes presenting findings in few studies. | Minor mistakes presenting findings in 2 studies. |
| **8. How precise are the results?** | Cannot tell.  (Most studies used descriptive statistics). | Yes, in some studies. | Yes, in most studies. |
| **9. Do you believe the results?** | See item 7. | See item 7. | Yes |
| **10. Can the results be applied to the local population?** | Cannot tell.  (Most studies used descriptive statistics Sample size was small in some studies). | To same extent. | Yes. |
| **11. Do the results of this study fit with other available evidence?** | Yes | Yes | Yes |
| **12. What are the implications of this study for practice?** | Cannot tell.  (Most studies used descriptive statistics. Sample size was small in some studies). | To same extent. | Yes |
| **Studies** | Acho et al. 2011.  Balestena et al. 2006; Bhadade et al. 2012; Blanco et al. 2016; Briones et al. 2015.  Chawla et al. 2015.  Estrada Altamirano. A. et al. 2002.  Fadiloglu et al. 2019; Fong et al. 2020; Franco-Sansaloni et al. 2017.  Garcia et al. 2009; Ghike et al. 2012; Gupta et al. 2011; Gupta et al. 2021.  Hernandez et al. 2020.  Jayaratnam et al. 2021.  Khergade et al. 2020; Kumar et al. 2021  Malpica et al. 2009; Montoya et al. 2011.  Okafor et al. 2014.  Orsini et al. 2011.  Osinaike et al. 2006.  Paumier at al.2020  Prats et al. 2011.  Prin at al. 2019.  Raad et al. 2003.  Rathod et al. 2016.  Richa et al. 2008.  Sailaja et al. 2019.  Sheela et al. 2004.  Shrestha et al. 2017.  Silva et al. 2020.  Thakur et al 2016.  Togal et al. 2010.  Tripathi et al. 2000.  Urbay et al 2002.  Vieira et al. 2016.  Zorrilla et al. 2017. | Acevedo et al. 2012; Afessa et al. 2001; Aldawood et al. 2011; Ayala et al. 2020 : Al-Jabari et al. 2001; Al Suleiman et al. 2006; Alves et al. 2021; Anwari et al. 2004; Ashraf et al. 2014; Bajwa et al. 2010; Baloch et al. 2010; Bandeira et al. 2014; Bentata et al. 2012; Bibi et al. 2008; Cabezas et al. 2004; Cheng et al. 2003; Cohen et al. 2000; Crozier et al. 2011; Dávila Gómez, H. L. et al. 2013; Dasgupta et al. 2017; De Greve et al. 2016; Dermikiran et al. 2003; Diaz et al. 2006; Farr et al. 2017; Farzi et al. 2017; Fouly et al. 2018; Galvez et al. 2009; Gilbert et al. 2003; Gombar et al. 2014; Gonzales et al. 2015; Hasbun et al. 2013; Joseph et al. 2018; Kallur et al. 2014; Igbaruma et al. 2016; Jain et al. 2016; Karolinski et al 2010; Keizer et al. 2006; Krawczyk et al. 2021; Lapinsky et al. 2011; Lataifeh et al 2010; Lawton et al. 2010; Leung et al. 2010; Lin et al. 2019; Lotufo et al. 2012; Loverro et al. 2001; Malvino et al. 2014; Miglani et al. 2020; Mirghani et al. 2004; Montes et al. 2018; Muench et al. 2008; Munnur et al. 2005; Murphy et al. 2002; Nava et al. 2016; Ng et al. 2014; Ngene et al. 2013; Oliveira et al. 2019; Oliveira S et al. 2019; Ozumba et al. 2018; Panda et al. 2018; Paternina et al. 2015; Paxton et al. 2014; Perez et al. 2008; Porreco et al. 2010; Quah et al. 2001; Qureshi et al. 2016; Ramachandra et al. 2013; Ramlakham et al. 2021; Rios et al. 2012; Rojas et al. 2011; Rojas JA, Cogollo, et al. 2011b; Rottenstreich et al. 2020 : Rudakemwa et al. 2021; Ryan et al. 2017; Sadler et al. 2013; Saif et al. 2013; Scarlett et al. 2009; Selo-Ojeme et al. 2005; Seppanen et al. 2016; Shaikh et al. 2013; Simsek et al. 2011; Small et al. 2012; Sriram et al. 2008; Stevens et al. 2006; Sultan et al. 2017;Taylor et al. 2000; Tempe et al. 2007; Thakur et al 2015; Vargas et al. 2019; Vasquez et al. 2007; Vasquez et al. 2014; Yi et al. 2018; Yousuf N. et al 2015; Yuel et al. 2008; Yuqui et al. 2017; Zhao et al. 2018. | Aoyama et al. 2019.  Barry et al. 2018.  Chantry et al. 2021.  Chantry et al. 2015.  Donati et al. 2012.  Estrada et al. 2021.  Godeberge et al. 2021.  Harrison et al. 2005.  Hazelgrove et al. 2011.  Jardine et al. 2021.  Karnad et al. 2004.  Madan et al 2009.  Maiden at el. 2020.  Mjahed et al. 2006.  Oliveira et al. 2009.  Oud 2017  Panchal et al. 2000.  Rossi et al. 2019.  Simpson et al. 2020.  Vasquez et al. 2015.  Wanderer et al. 2013.  Zwart et al. 2010. |

CASP: Critical Appraisal Skills Programme. IPV: Intimate partner violence.
