# Supplementary material for: Social determinants and exposure to intimate partner violence in women with severe acute maternal morbidity in the intensive care unit: a systematic review
Source: BMC Pregnancy Childbirth. 2023 Sep 12;23:656. doi: 10.1186/s12884-023-05927-5 (PMC10496274; doi:10.1186/s12884-023-05927-5)
Supplement: Supplementary file 4 — Additional file 4: Supplementary Appendix S4. List of included studies on women with severe acute maternal morbidity in the intensive care unit. [file 12884_2023_5927_MOESM4_ESM.docx]

**Supplementary Appendix S4**

**List of included studies on women with severe acute maternal morbidity in the intensive care unit**

**(references supporting Table 2).**

1. Igbaruma, S., Olagbuji, B., Aderoba, A., Kubeyinje, W., Ande, B., & Imarengiaye, C. (2016). Severe maternal morbidity in a general intensive care unit in Nigeria: clinical profiles and outcomes. *International Journal of Obstetric Anesthesia*, 28, 39-44. doi: 10.1016/j.ijoa.2016.07.008
2. Lotufo, F. A., Parpinelli, M. A., Haddad, S. M., Surita, F. G., & Cecatti, J. G. (2012). Applying the new concept of maternal near-miss in an intensive care unit. *Clinics*, 67(3), 225-230. doi:10.6061/clinics/2012(03)04
3. Nava, M. L., Silva Bentacourt, Á., Labarca, L., Urdaneta M, J. R., González I, M. E., Contreras Benítez, A., . . . Fernández, M. (2016). Caracterización de la paciente obstétrica críticamente enferma, experiencia de la maternidad "Dr. Armando Castillo Plaza", Maracaibo, Venezuela: 2011 - 2014. *Revista Chilena de Obstetricia y Ginecología*, 81(4), 288 - 296. doi:10.4067/S0717-75262016000400004
4. Selo-Ojeme, D. O., Omosaiye, M., Battacharjee, P., & Kadir, R. A. (2005). Risk factors for obstetric admissions to the intensive care unit in a tertiary hospital: A case-control study. *Archives of Gynecology and Obstetrics*, 272(3), 207-210. doi:10.1007/s00404-004-0695-x
5. Small, M. J., James, A. H., Kershaw, T., Thames, B., Gunatilake, R., & Brown, H. (2012). Near-miss maternal mortality: cardiac dysfunction as the principal cause of obstetric intensive care unit admissions. *Obstetrics & Gynecology*, 119(2 Pt 1), 250-255.doi: 10.1097/AOG.0b013e31824265c7
6. Panchal, S., Arria, A. M., & Harris, A. P. (2000). Intensive care utilization during hospital admission for delivery: Prevalence, risk factors, and outcomes in a statewide population. *Anesthesiology*, 92(6), 1537-1544. doi:10.1097/00000542-200006000-00009
7. Madan, I., Puri, I., Jain, N. J., Grotegut, C., Nelson, D., & Dandolu, V. (2009). Characteristics of obstetric intensive care unit admissions in New Jersey. *Journal of Maternal-Fetal & Neonatal Medicine*, 22(9), 785-790. doi:10.3109/14767050902874097
8. Gupta, S., Naithani, U., Doshi, V., Bhargava, V., & Vijay, B. S. (2011). Obstetric critical care: A prospective analysis of clinical characteristics, predictability, and fetomaternal outcome in a new dedicated obstetric intensive care unit. *Indian Journal of Anaesthesia*, 55(2), 146-153. doi:10.4103/0019-5049.79895
9. Bajwa, S. K., Bajwa, S. J., Kaur, J., Singh, K., & Kaur, J. (2010). Is intensive care the only answer for high risk pregnancies in developing nations? *Journal of Emergencies, Trauma and Shock*, 3(4), 331-336. doi:10.4103/0974-2700.70752
10. Bibi, S., Memon, A., Sheikh, J. M., & Qureshi, A. H. (2008). Severe acute maternal morbidity and intensive care in a public sector university hospital of Pakistan. *Journal of Ayub Medical College, Abbottabad: JAMC*, 20(1), 109-112.
11. Paternina-Caicedo, A. J., Rojas-Suarez, J. A., Dueñas-Castel, C., Miranda-Quintero, J. E., & Bourjeily, G. (2015). Mortality risk prediction with an updated acute physiology and chronic health evaluation II score in critically ill obstetric patients: A cohort study. *Journal of Intensive Care Medicine*, 30(2), 97-102. doi:10.1177/0885066613502450
12. Rojas, J. A., Cogollo, M., Miranda, J. E., Ramos, E. C., Fernández, J. C., & Bello, A. M. (2011). Morbilidad materna extrema en cuidados intensivos obstétricos: Cartagena (Colombia) 2006 - 2008. *Revista Colombiana de Obstetricia y Ginecologia*, 62(2), 131-140. doi:10.18597/rcog.223
13. Saif, K. M., Tahmina, S., & Maitree, P. (2013). A prospective study of clinical profile and outcome of critically ill obstetric patients in ICU at a tertiary level hospital in India. *Anaesthesia, Pain & Intensive Care*, 17(3), 243-247.
14. Osinaike, B. B., Amanor-Boadu, S. D., & Sanusi, A. A. (2006). Obstetric intensive care: a developing country experience. *The Internet Journal of Anesthesiology*, 10(2), 1 - 5.
15. Vasquez, D. N., Das Neves, A. V., Vidal, L., Moseinco, M., Lapadula, J., Zakalik, G., . . . Estenssoro, E. (2015). Characteristics, Outcomes, and Predictability of Critically Ill Obstetric Patients: A Multicenter Prospective Cohort Study. *Critical Care Medicine*, 43(9): 1887-1897. doi:10.1097/ccm.0000000000001139
16. Jain, S., Guleria, K., Suneja, A., Vaid, N. B., & Ahuja, S. (2016). Use of the Sequential Organ Failure Assessment score for evaluating outcome among obstetric patients admitted to the intensive care unit. *International Journal of Gynaecology and Obstetrics*, 132(3), 332-336. doi:10.1016/j.ijgo.2015.08.005
17. Ramachandra Bhat, P. B., Navada, M. H., Rao, S. V., & Nagarathna, G. (2013). Evaluation of obstetric admissions to intensive care unit of a tertiary referral center in coastal India. *Indian Journal of Critical Care Medicine*, 17(1), 34-37. doi:10.4103/0972-5229.112156
18. Acevedo Rodríguez, O., Sáez Cantero, V., Pérez Assef, A., & Alcina Pereira, S. (2012). Caracterización de la morbilidad materna severa en una Unidad de Cuidados Intensivos. Revista Cubana de Ginecologia y Obstetricia, 38(2), 148-160.
19. Montoya Cid, F. R., León Cid, I., Hernández Pedroso, W., Segura Fernández, A., Rodríguez Iglesias, G., & Atienza Barzaga, A. M. (2011). Caracterización de pacientes obstétricas críticas. *Revista Cubana de Medicina Militar*, 40(2), 126-136.
20. Perez, A., Bacallao, J., Alcina, S., & Gomez, Y. (2008). Severe maternal morbidity in the intensive care unit of a havana teaching hospital, 1998 to 2004. *MEDICC Review*, 10(3), 17-23. doi: 10.37757/MR2008.V10.N3.7
21. Taylor, R., & Richards, G. A. (2000). Critically ill obstetric and gynaecological patients in the intensive care unit. *South African Medical Journal*, 90(11), 1140-1144.
22. Paxton, J. L., Presneill, J., & Aitken, L. (2014). Characteristics of obstetric patients referred to intensive care in an Australian tertiary hospital. *Australian and New Zealand Journal of Obstetrics and Gynaecology*, 54(5), 445-449. doi:10.1111/ajo.12211
23. Leung, N. Y. W., Lau, A. C. W., Chan, K. K. C., & Yan, W. W. (2010). Clinical characteristics and outcomes of obstetric patients admitted to the Intensive Care Unit: A 10-year retrospective review. *Hong Kong Medical Journal*, 16(1), 18-25.
24. Ng, V. K. S., Lo, T. K., Tsang, H. H., Lau, W. L., & Leung, W. C. (2014). Intensive care unit admission of obstetric cases: A single centre experience with contemporary update. *Hong Kong Medical Journal*, 20(1), 24-31. doi:10.12809/hkmj133924
25. Donati, S., Senatore, S., Ronconi, A., & the Regional Maternal Mortality Working, G. (2012). Obstetric near-miss cases among women admitted to intensive care units in Italy. *Acta Obstetricia et Gynecologica Scandinavica*, 91(4), 452-457. doi:10.1111/j.1600-0412.2012.01352.x
26. Keizer, J. L., Zwart, J. J., Meerman, R. H., Harinck, B. I. J., Feuth, H. D. M., & van Roosmalen, J. (2006). Obstetric intensive care admissions: A 12-year review in a tertiary care centre. *European Journal of Obstetrics & Gynecology and Reproductive Biology, 128*(1–2), 152-156. doi:[10.1016/j.ejogrb.2005.12.013](http://dx.doi.org/10.1016/j.ejogrb.2005.12.013)
27. Zwart, J. J., Dupuis, J. R., Richters, A., Ory, F., & van Roosmalen, J. (2010). Obstetric intensive care unit admission: a 2-year nationwide population-based cohort study. *Intensive Care Medicine*, 36(2), 256-263. doi:10.1007/s00134-009-1707-x
28. Lawton, B. A., Wilson, L. F., Dinsdale, R. A., Rose, S. B., Brown, S. A., Tait, J., . . . McCaw, A. (2010). Audit of severe acute maternal morbidity describing reasons for transfer and potential preventability of admissions to ICU. *Australian & New Zealand Journal of Obstetrics & Gynaecology*, 50(4), 346-351. doi:10.1111/j.1479-828X.2010.01200.x
29. Sadler, L. C., Austin, D. M., Masson, V. L., McArthur, C. J., McLintock, C., Rhodes, S. P., & Farquhar, C. M. (2013). Review of contributory factors in maternity admissions to intensive care at a New Zealand tertiary hospital. *American Journal of Obstetrics and Gynecology*, 209(6), 549.e541-549.e547. doi:10.1016/j.ajog.2013.07.031
30. Cheng, C., & Raman, S. (2003). Intensive care use by critically ill obstetric patients: a five-year review. *International Journal of Obstetric Anesthesia,* 12(2), 89-92. doi: 10.1016/S0959-289X(02)00154-1
31. Quah, T. C. C., Chiu, J. W., Tan, K. H., Yeo, S. W., & Tan, H. M. (2001). Obstetric admissions to the intensive therapy unit of a tertiary care institution. *Annals of the Academy of Medicine*, Singapore, 30(3), 250-253.
32. Wanderer, J. P., Leffert, L. R., Mhyre, J. M., Kuklina, E. V., Callaghan, W. M., & Bateman, B. T. (2013). Epidemiology of obstetric-related ICU admissions in Maryland: 1999-2008. *Critical Care Medicine*, 41(8), 1844-1852. doi:10.1097/CCM.0b013e31828a3e24
33. Afessa, B., Green, B., Delke, I., & Koch, K. (2001). Systemic inflammatory response syndrome, organ failure, and outcome in critically ill obstetric patients treated in an ICU. *Chest*, 120(4), 1271-1277. doi: 10.1378/chest.120.4.1271
34. Thakur, M., Gonik, B., Gill, N., Awonuga, A. O., Rocha, F. G., & Gonzalez, J. M. (2016). Intensive Care Admissions in Pregnancy: Analysis of a Level of Support Scoring System. *Maternal & Child Health Journal*, 20(1), 106-113. doi: 10.1007/s10995-015-1808-9
35. Gilbert, T. T., Smulian, J. C., Martin, A. A., Ananth, C. V., Scorza, W., & Scardella, A. T. (2003). Obstetric admissions to the intensive care unit: Outcomes and severity of illness. *Obstetrics & Gynecology*, 102(5), 897-903. doi:10.1016/S0029-7844(03)00767-1
36. Kallur, S. D., Patil Bada, V., Reddy, P., Pandya, S., & Nirmalan, P. K. (2014). Organ dysfunction and organ failure as predictors of outcomes of severe maternal morbidity in an obstetric intensive care unit. *Journal of Clinical & Diagnostic Research*, 8(4), Oc06-08. doi:10.7860/jcdr/2014/8068.4213
37. Aldawood, A. (2011). Clinical characteristics and outcomes of critically ill obstetric patients: a ten-year review. *Annals of Saudi Medicine*, 31(5), 518-522. doi:10.4103/0256-4947.84631
38. Anwari, J. S., Butt, A. A., & Al-Dar, M. A. (2004). Obstetric admissions to the intensive care unit. *Saudi Medical Journal*, 25(10), 1394-1399.
39. Munnur, U., Karnad, D. R., Bandi, V. D., Lapsia, V., Suresh, M. S., Ramshesh, P., . . . Guntupalli, K. K. (2005). Critically ill obstetric patients in an American and an Indian public hospital: comparison of case-mix, organ dysfunction, intensive care requirements, and outcomes. *Intensive Care Medicine*, 31(8), 1087-1094. doi:10.1007/s00134-005-2710-5
40. Acho-Mego, S. C., Salvador Pichilingue, J., Díaz-Herrera, J. A., & García-Meza, M. (2011). Morbilidad materna extrema: admisiones ginecoobstétricas en las unidad es de cuidad os intensivos de un hospital general. *Revista Peruana de Ginecología y Obstetricia*, 57, 87 - 92. doi:10.31403/rpgo.v57i190
41. Al-Jabari, A. S., Al-Meshari, A. A., Takrouri, M. S., & Seraj, M. A. (2001). Gynecology and obstetrical conditions requiring intensive care admission. *Saudi Medical Journal*, 22(11), 980-983.
42. Al-Suleiman, S., Qutub, H., Rahman, J., & Rahman, M. S. (2006). Obstetric admissions to the intensive care unit: a 12-year review. *Archives of Gynecology and Obstetrics*, 274(1), 4-8. doi:10.1007/s00404-004-0721-z
43. Ashraf, N., Mishra, S. K., Kundra, P., Veena, P., Soundaraghavan, S., & Habeebullah, S. (2014). Obstetric patients requiring intensive care: A one year retrospective study in a tertiary care institute in India. *Anesthesiology Research and Practice*, 2014. doi:10.1155/2014/789450
44. Balestena Sánchez, J. M., Márquez Suárez, D., Pastrana Roman, I., Fernández Alech, R., & Pérez Labrador, J. (2006). Algunos aspectos epidemiológicos en la morbilidad obstétrica crítica. Some epidemiological aspects in critical obstetric morbidity. *Revista de Ciencias Medicas de Pinar del Rio*, 10(3), 51-60. 45.
45. Baloch, R., Jakhrani, N., Zeb, E., Hafeez, S., Abassi, M., & Abbasi, F. (2010). Pattern and outcome of obstetric admissions to the surgical intensive care unit - a ten years study. *Journal of Surgery Pakistan*, 15(4), 171 - 176.
46. Bandeira, A. R. A. P., Rezende, C. A. L., Reis, Z. S. N., Barbosa, A. R., Peret, F. J. A., & Cabral, A. C. V. (2014). Epidemiologic profile, survival, and maternal prognosis factors among women at an obstetric intensive care unit. *International Journal of Gynecology and Obstetrics*, 124(1), 63-66. doi:10.1016/j.ijgo.2013.07.015
47. Bentata, Y., Housni, B., Mimouni, A., & Abouqal, R. (2012). Admissions of women in the third trimester of pregnancy to an intensive care unit in Morocco over a 4-year period. *International Journal of Gynaecology & Obstetrics,* 116(3), 260-261. doi:10.1016/j.ijgo.2011.10.021
48. Bhadade, R., De' Souza, R., More, A., & Harde, M. (2012). Maternal outcomes in critically ill obstetrics patients: A unique challenge. *Indian Journal of Critical Care Medicin*e, 16(1), 8-16. doi:10.4103/0972-5229.94416
49. Blanco Esquivel, L. A., Urbina, J. M., & Zerón, H. M. (2016). Approach to an obstetric prognosis scale: The modified SOFA scale. *Ghana Medical Journal*, 50(3), 129-135.
50. Briones Garduño, J. C., Viruez Soto, J. A., Vallejo Narváez, C. M., Vargas Arias, R. E., Ortiz Bolaños, R., & Díaz de León Ponce, M. A. (2015). Aislamientos microbiológicos: experiencia en obstetricia crítica. *Revista de la Asociación Mexicana de Medicina Crítica y Terapia Intensiva*, 29(4), 209-213.
51. Cabezas Poblet, B., Valle Martínez, Y., Cabezas Poblet, M., Hernández Barrios, E., & Espín González, R. (2004). Caracterización de la paciente obstétrica en estado crítico en la provincia de Cienfuegos. *Medisur (Revista Electronica de las Ciencias Médicas en Cienfuegos)*, 2(1), 11 - 16.
52. Chantry, A. A., Deneux-Tharaux, C., Bonnet, M. P., & Bouvier-Colle, M. H. (2015). Pregnancy-related ICU admissions in France: Trends in rate and severity, 2006-2009. *Critical Care Medicine*, 43(1), 78-86. doi:10.1097/CCM.0000000000000601
53. Chawla, S., Nakra, M., Mohan, S., Nambiar, B. C., Agarwal, R., & Marwaha, A. (2013). Why do obstetric patients go to the ICU? A 3-year-study. *Medical Journal Armed Forces India*, 69(2), 134-137. doi:10.1016/j.mjafi.2012.08.033
54. Cohen, J., Singer, P., Kogan, A., Hod, M., & Bar, J. (2000). Course and outcome of obstetric patients in a general intensive care unit. *Acta Obstetricia et Gynecologica Scandinavica*, 79(10), 846-850. doi:10.1034/j.1600-0412.2000.079010846.x
55. Crozier, T. M., & Wallace, E. M. (2011). Obstetric admissions to an integrated general intensive care unit in a quaternary maternity facility. *Australian and New Zealand Journal of Obstetrics and Gynaecology*, 51(3), 233-238. doi:10.1111/j.1479-828X.2011.01303.x
56. Dávila Gómez, H. L., Rodríguez Matos, M., Peña Martínez, M. L., García Valdés, A., Rueda Rodríguez, R., & Matos Rodríguez, Z. (2013). Morbilidad de la paciente obstétrica extremadamente grave en la Isla de la Juventud, 2002-2010. *Progresos de Obstetricia y Ginecologia*, 56(6), 310-315. doi: 10.1016/j.pog.2013.01.001
57. De Greve, M., Van Mieghem, T., Van Den Berghe, G., & Hanssens, M. (2016). Obstetric Admissions to the Intensive Care Unit in a Tertiary Hospital. *Gynecologic and Obstetric Investigation*, 81(4), 315-320. doi:10.1159/000431224
58. Demirkiran, O., Dikmen, Y., Utku, T., & Urkmez, S. (2003). Critically ill obstetric patients in the intensive care unit. *International Journal of Obstetric Anesthesia*, 12(4), 266-270. doi:10.1016/S0959-289X(02)00197-8
59. Dıaz Mayo, J., Perez Aseef, A., & Naranjo Igarza, S. (2006). Morbilidad materna en la Unidad de Cuidados Intensivos en el Hospital General Docente Enrique Cabrera. *Revista Cubana de Obstetricia y Ginecologia*, 3(45 - 9).
60. Estrada Altamirano, A., Hernández Pacheco, J. A., Cisneros Castelo, M., & Quesnel García, C. (2002). Experiencia de la unidad de cuidados intensivos obstétricos del Instituto Nacional de Perinatología, 1993-1998. *Perinatologia y Reproduccion Humana*, 16(2), 85-95.
61. Gálvez-Vengoechea, M. L., Arreaza-Graterol, M., & Rodríguez-Ortiz, J. A. (2009). Mortalidad materna de pacientes atendidas en la UCI del Hospital Simón Bolívar, Bogotá (Colombia) 2004 - 2006. *Revista Colombiana de Obstetricia y Ginecologia*, 60(2), 152-158. doi: 10.18597/rcog.340
62. García López, M., Ontiveros Morales, M. P., & Whizar-Lugo, V. M. (2009). Admisiones Obstétrica en la Unidad de Cuidados Intensivos de un Hospital Comunitario. *Anestesia en México*, 21(1), 7 - 11.
63. Ghike, S., & Asegaonkar, P. (2012). Why Obstetric Patients are admitted to Intensive Care Unit? A Retrospective Study. Journal of South Asian Federation of Obstetrics and Gynaecology, 4(2), 90-92. doi: 10.5005/jp-journals-10006-1181
64. Gombar, S., Ahuja, V., & Jafra, A. (2014). A retrospective analysis of obstetric patient's outcome in intensive care unit of a tertiary care center. *Journal of Anaesthesiology Clinical Pharmacology*, 30(4), 502-507. doi:10.4103/0970-9185.142843
65. González Aguilera, J. C., Vázquez Belizón, Y. E., Pupo Jiménez, J. M., Algas Hechavarría, L. A., & Cabrera Lavernia, J. O. (2015). Morbilidad materna extrema en una unidad de cuidados intensivos. *MEDISAN*, 19(12), 1466-1476.
66. Harrison, D., Penny, J., Yentis, S. M., Fayek, S., & Brady, A. (2005). Case mix,outcome and activity for obstetric admissions to adult, general critical care units: a secondary analysis of the ICNARC Case Mix Programme Database. *Critical Care*, 9, S25-37. doi: 10.1186/cc3542
67. Hasbun, H. J., Sepulveda-Martinez, A., Cornejo, R. R., & Romero, P. C. (2013). Intensive care admissions due to severe maternal morbidity. *Revista Medica de Chile*, 141(12), 1512-1519. doi: 10.4067/S0034-98872013001200003
68. Hazelgrove, J. F., Price, C., Pappachan, V. J., & Smith, G. B. (2001). Multicenter study of obstetric admissions to 14 intensive care units in southern England. *Critical Care Medicine*, 29(4), 770-775. doi: 10.1097/00003246-200104000-00016
69. Karnad, D. R., Lapsia, V., Krishnan, A., & Salvi, V. S. (2004). Prognostic factors in obstetric patients admitted to an Indian intensive care unit. Critical Care Medicine, 32(6), 1294-1299. doi:10.1097/01.CCM.0000128549.72276.00
70. Karolinski, A., Mazzoni, A., Belizan, J. M., Althabe, F., Bergel, E., & Buekens, P. (2010). Lost opportunities for effective management of obstetric conditions to reduce maternal mortality and severe maternal morbidity in Argentina and Uruguay. *International Journal of Gynecology and Obstetrics*, 110(2), 175-180. doi:http://dx.doi.org/10.1016/j.ijgo.2010.05.002
71. Lapinsky, S. E., Hallett, D., Collop, N. A., Drover, J., Lavercombe, P., Leeman, M., . . . Moodley, J. (2011). Evaluation of standard and modified severity of illness scores in the obstetric patient. *Journal of Critical Care*, 26(5), 535.e531-537. doi:10.1016/j.jcrc.2010.10.003
72. Lataifeh, I., Amarin, Z., Zayed, F., Al-Mehaisen, L., Alchalabi, H., & Khader, Y. (2010). Indications and outcome for obstetric patients' admission to intensive care unit: a 7-year review. *Journal of Obstetrics and Gynaecology*, 30(4), 378-382. doi: 10.3109/01443611003646298
73. Loverro, G., Pansini, V., Greco, P., Vimercati, A., Parisi, A. M., & Selvaggi, L. (2001). Indications and outcome for intensive care unit admission during puerperium. *Archives of Gynecology and Obstetrics*, 265(4), 195-198. doi:10.1007/s004040000160
74. Malpica Alonso, E., Ruesca Domınguez, C., Perez Silva, M., Garcıa Malpica, K., & K., M. (2008). Comportamiento de la paciente obstetrica grave. Experiencias de cuatro anos de trabajo. *Revista Medica Electronica*, 30(3).
75. Malvino, E. (2014). Morbilidad materna aguda severa y condiciones de gravedad de enfermas obstétricas al ingreso en una Unidad de Cuidados Intensivos. *Revista Argentina de Terapia Intensiva*, 31(4).
76. Mirghani, H. M., Hamed, M., Ezimokhai, M., & Weerasinghe, D. S. L. (2004). Pregnancy-related admissions to the intensive care unit. *International Journal of Obstetric Anesthesia*, 13(2), 82-85. doi:10.1016/j.ijoa.2003.10.004
77. Mjahed, K., Hamoudi, D., Salmi, S., & Barrou, L. (2006). Obstetric patients in a surgical intensive care unit: prognostic factors and outcome. *Journal of Obstetrics and Gynaecology*, 26(5), 418-423. doi:10.1080/01443610600720188
78. Muench, M. V., Baschat, A. A., Malinow, A. M., & Mighty, H. E. (2008). Analysis of disease in the obstetric intensive care unit at a university referral center. *Journal of Reproductive Medicine*, 53(12), 914-920.
79. Murphy, D. J., & Charlett, P. (2002). Cohort study of near-miss maternal mortality and subsequent reproductive outcome. *European Journal of Obstetrics & Gynecology and Reproductive Biology*, 102 (2), 173-178. doi: 10.1016/s0301-2115(01)00320-7
80. Ngene, N. C., Moodley, J., Songca, P., von Rahden, R., Paruk, F., Onyia, C. O., & van der Linde, S. (2013). Maternal and fetal outcomes of HIV-infected and non-infected pregnant women admitted to two intensive care units in Pietermaritzburg, South Africa. *South African Medical Journal*, 103(8), 543-548. doi:10.7196/samj.6590
81. Okafor, U. V., & Aniebue, U. (2004). Admission pattern and outcome in critical care obstetric patients. *International Journal of Obstetric Anesthesia*, 13(3), 164-166. doi: 10.1016/j.ijoa.2004.04.002
82. Oliveira-Neto, A. F., Parpinelli, M. A., Cecatti, J. G., Souza, J. P., & Sousa, M. H. (2009). Factors associated with maternal death in women admitted to an intensive care unit with severe maternal morbidity. *International Journal of Gynecology and Obstetrics*, 105(3), 252-256. doi:10.1016/j.ijgo.2009.01.025
83. Orsini, J., Butala, A., Diaz, L., Muzylo, E., Mainardi, C., & Kastell, P. (2012). Clinical Profile of Obstetric Patients Admitted to the Medical-Surgical Intensive Care Unit (MSICU) of an Inner-City Hospital in New York. *Journal of Clinical Medical Research, 4*(5), 314-317. doi:10.4021/jocmr1079w
84. Porreco, R. P., & Barkey, R. (2010). Peripartum intensive care. *Journal of Maternal-Fetal & Neonatal Medicine*, 23(10), 1136-1138. doi:10.3109/14767058.2010.490890
85. Prats Álvarez, O. M., Casanova Moreno, M. d. l. C., Regalado Carvajal, A. J., Doris Yadira, D. D., Doris Yadira, & Rivera Martínez, M. E. (2011). Morbilidad y mortalidad de la materna grave. Pinar del Río 2000-2010. Revista Ciencias Medicas de Pinar del Rio, 15(2), 133-144.
86. Qureshi, R., Irfan Ahmed, S., Raza, A., Khurshid, A., & Chishti, U. (2016). Obstetric patients in intensive care unit: Perspective from a teaching hospital in Pakistan. *Journal of the Royal Society of Medicine Open*, 7(11), 2054270416663569. doi:10.1177/2054270416663569
87. Raad López, A. F., Pérez Ojeda, R., Ávila Batista, S., & Rodríguez Pompa, Y. (2003). Comportamiento de Morbilidad Materna en el Hospital Dr Ernesto Guevara de Las Tunas 1998 2000. *Revista electrónica Dr. Zoilo E. Marinello Vidaurreta*, 21.
88. Rathod, A. T., & Malini, K. V. (2016). Study of Obstetric Admissions to the Intensive Care Unit of a Tertiary Care Hospital. *The Journal of Obstetrics and Gynecology of India*, 66(Suppl 1), 12-17. doi: 10.1007/s13224-015-0750-589.
89. Richa, F., Karim, N., & Yazbeck, P. (2008). Obstetric admissions to the intensive care unit: an eight-year review. *Lebanese Medical Journal*, 56(4), 215-219.
90. Rios, F. G., Risso-Vázquez, A., Alvarez, J., Vinzio, M., Falbo, P., Rondinelli, N., & Bienzobas, D. H. (2012). Clinical characteristics and outcomes of obstetric patients admitted to the intensive care unit. *International Journal of Gynecology and Obstetrics*, 119(2), 136-140. doi:10.1016/j.ijgo.2012.05.039
91. Rojas, J. A., Miranda, J. E., Ramos, E., & Fernández, J. C. (2011). Cuidado crítico en la paciente obstétrica. Complicaciones, intervenciones y desenlace maternofetal. *Clinica e Investigacion en Ginecologia y Obstetrica,* 38(2), 44-49. doi:10.1016/j.gine.2009.10.004
92. Scarlett, M., Isaacs, M.-A., Fredrick-Johnston, S., Kulkarni, S., McCaw-Binns, A., & Fletcher, H. (2009). Maternal mortality in patients admitted to an intensive care unit in Jamaica. *International Journal of Gynecology and Obstetrics*, 105(2), 169-170. doi:http://dx.doi.org/10.1016/j.ijgo.2008.12.007
93. Seppänen, P., Sund, R., Roos, M., Unkila, R., Meriläinen, M., Helminen, M., . . . Suominen, T. (2016). Obstetric admissions to ICUs in Finland: A multicentre study. *Intensive & Critical Care Nursing,* 35, 38-44. doi: 10.1016/j.iccn.2016.03.002. This study complement data with Seppänen, P. M., Sund, R. T., Uotila, J. T., Helminen, M. T., & Suominen, T. M. (2020). Maternal and neonatal characteristics in obstetric intensive care unit admissions. International Journal of Obstetric Anesthesia, 41, 65-70. doi:10.1016/j.ijoa.2019.07.002 (the latter study showed data on parturient and infant).
94. Shaikh, S., Shaikh, N., Abassi, R., & Balouch, R. (2013). Obstetric admission to the intensive care unit: a one year review. *Medical Channel*, 19(3), 59 - 63.
95. Sheela, C. N., Mhaskar, A., & Mhaskar, R. (2004). Critical care in obstetrics-A 3 year review in a tertiary referral hospital. *Journal of Obstetrics and Gynecology of India*, 54, 155-157.
96. Şimşek, T., Eyigör, C., Uyar, M., Karaman, S., & Moral, A. R. (2011). Retrospective review of critically ill obstetrical patients: A decade's experience. *Turkish Journal of Medical Sciences*, 41(6), 1059-1064. doi:10.3906/sag-1009-5
97. Sriram, S., & Robertson, M. S. (2008). Critically ill obstetric patients in Australia: a retrospective audit of 8 years' experience in a tertiary intensive care unit. *Critical Care and Resuscitation: Journal of the Australasian Academy of Critical Care Medicine*, 10(2), 124.
98. Stevens, T. A., Carroll, M. A., Promecene, P. A., Seibel, M., & Monga, M. (2006). Utility of Acute Physiology, Age, and Chronic Health Evaluation (APACHE III) score in maternal admissions to the intensive care unit. *American Journal of Obstetrics and Gynecology*, 194(5), e13-15. doi:10.1016/j.ajog.2006.01.073
99. Tempe, A., Wadhwa, L., Gupta, S., Bansal, S., & Satyanarayana, L. (2007). Prediction of mortality and morbidity by simplified acute physiology score II in obstetric intensive care unit admissions. *Indian Journal of Medical Sciences*, 61(4), 179-185. doi: 10.4103/0019-5359.31151
100. Thakur A, Basnet P, Agrawal A, Uprety DK. (2015). Profile of Patients Admitted in Maternal Intensive Care Unit at BPKIHS, a Tertiary Hospital in Eastern Nepal. *Journal of Nepal Health Research Council*,13(29):90-4.
101. Togal, T., Yucel, N., Gedik, E., Gulhas, N., Toprak, H. I., & Ersoy, M. O. (2010). Obstetric admissions to the intensive care unit in a tertiary referral hospital. Journal of Critical Care, 25(4), 628-633. doi:10.1016/j.jcrc.2010.02.015
102. Tripathi, R., Rathore, A. M., & Saran, S. (2000). Intensive care for critically ill obstetric patients. International Journal of Gynecology and Obstetrics, 68(3), 257-258. doi: 10.1016/S0020-7292(99)00200-3
103. Urbay Ruiz, A. A., Cruz Martínez, H., Fong Seuc, L., Santos Pérez, L. A., Valledor Tristá, R., & Molerio Sáez, L. E. (2002). Comportamiento de la enfermedad obstétrica grave. Nueve Años en una Unidad de Cuidados Intensivos. *Medicentro*, 6, 1-8.
104. Vasquez, D. N., Das Neves, A. V., Aphalo, V. B., Loudet, C. I., Roberti, J., Cicora, F., . . . Estenssoro, E. (2014). Health insurance status and outcomes of critically ill obstetric patients: a prospective cohort study in Argentina. *Journal of Critical Care*, 29(2), 199-203. doi:10.1016/j.jcrc.2013.11.010
105. Vasquez, D. N., Estenssoro, E., Canales, H. S., Reina, R., Saenz, M. G., Das Neves, A. V., . . . Loudet, C. I. (2007). Clinical characteristics and outcomes of obstetric patients requiring ICU admission. *Chest*, 131(3), 718-724. doi:10.1378/chest.06-2388
106. Vieira Saintrain, S., Gomes Ramalho de Oliveira, J., Vieira de Lima Saintrain, M., Vieira Bruno, Z. V., Nogueira Borges, J. L., De Francesco Daher, E., & Bezerra da Silva Jr, G. (2016). Factors associated with maternal death in an intensive care unit. *Revista Brasileira de Terapia Intensiva*, 28(4), 397-404. doi:10.5935/0103-507X.20160073
107. Yousuf, N., Shaikh, S. N., Soomro, A., & Baloch, R. (2015). Analysis of clinical characteristics, rationale, and management of critically ill obstetric patients transferred to ICU. *Journal of the Pakistan Medical Association,* 65(9), 959-962.
108. Yuel, V. Y., Kaur, V., Kaur, G., Andappan, A., & Afzal, L. (2008). Critical care in obstetrics – scenario in a developing. *The Journal of Obstetrics and Gynecology of India*, 58(3), 217-220.
109. Barry, Y., Deneux-Tharaux, C., Saucedo, M., Goulet, V., Guseva-Canu, I., Regnault, N., & Chantry, A. A. (2018). Maternal admissions to intensive care units in France: Trends in rates, causes and severity from 2010 to 2014. *Anaesthesia, Critical Care & Pain Medicine*. doi:10.1016/j.accpm.2018.12.007
110. Dasgupta, S., Jha, T., Bagchi, P., Singh, S. S., Gorai, R., & Choudhury, S. D. (2017). Critically Ill Obstetric Patients in a General Critical Care Unit: A 5 Years' Retrospective Study in a Public Teaching Hospital of Eastern India. Indian J Crit Care Med, 21(5), 294-302. doi:10.4103/ijccm.IJCCM_445_16
111. Farr, A., Lenz-Gebhart, A., Einig, S., Ortner, C., Holzer, I., Elhenicky, M., . . . Lehner, R. (2017). Outcomes and trends of peripartum maternal admission to the intensive care unit. *Wien Klin Wochenschr*, 129(17-18), 605-611. doi:10.1007/s00508-016-1161-z
112. Farzi, F., Mirmansouri, A., Atrkar Roshan, Z., Naderi Nabi, B., Biazar, G., & Yazdipaz, S. (2017). Evaluation of Admission Indications, Clinical Characteristics and Outcomes of Obstetric Patients Admitted to the Intensive Care Unit of a Teaching Hospital Center: A Five-Year Retrospective Review. *Anesthesilology and Pain Medicine*, 7(3), e13636. doi:10.5812/aapm.13636
113. Fouly, H., Abdou, F. A., Abbas, A. M., & Omar, A. M. (2018). Audit for quality of care and fate of maternal critical cases at Women's Health Hospital. *Applied Nursing Research*, 39, 175-181. doi:10.1016/j.apnr.2017.11.003
114. Franco-Sansaloni, A., Vizcaíno-Torres, J., Estelles-Morant, D., Villar-Graullera, E., & Serrano-Fernández, J. (2017). Morbilidad y mortalidad de pacientes obstétricas en una unidad de cuidados intensivos. Ginecologia y Obstetricia de Mexico, 85(1), 7-12.
115. Joseph, C. M., Bhatia, G., Abraham, V., & Dhar, T. (2018). Obstetric admissions to tertiary level intensive care unit - Prevalence, clinical characteristics and outcomes. *Indian Journal of Anaesthesia*, 62(12), 940-944. doi:10.4103/ija.IJA_537_18
116. Monte AS, Rocha Teles LM, Batista Oria MO, Costa Carvalho FH, Brown H, Kelve de castro D. Comparison between near miss criteria in a maternal intensive care unit. *Revista da Escola de Enfermagem da USP*. 2018; 52: e03404.
117. Oud, L. (2017). Epidemiology of Pregnancy-Associated ICU Utilization in Texas: 2001 - 2010. J Clin Med Res, 9(2), 143-153. doi:10.14740/jocmr2854w
118. Ozumba, B. C., Ajah, L. O., Obi, V. O., Umeh, U. A., Enebe, J. T., & Obioha, K. C. (2018). Pattern and Outcome of Obstetric Admissions into the Intensive Care Unit of a Southeast Nigerian Hospital. *Indian Journal of Critical Care Medicine: peer-reviewed, official publication of Indian Society of Critical Care Medicine*, 22(1), 16-19. doi:10.4103/ijccm.IJCCM_297_17
119. Ryan, H. M., Jones, M. A., Payne, B. A., Sharma, S., Hutfield, A. M., Lee, T., . . . von Dadelszen, P. (2017). Validating the Performance of the Modified Early Obstetric Warning System Multivariable Model to Predict Maternal Intensive Care Unit Admission. *Journal of Obstetrics & Gynaecology Canada: JOGC,* 39(9), 728-733.e723. doi:10.1016/j.jogc.2017.01.028
120. Shrestha, A., & Kayastha, B. (2017). A Study on Obstetric Intensive Care in Dhulikhel Hospital, Kathmandu University Hospital. Kathmandu University Medical Journal, 15(59), 240-243.
121. Sultan, E. A., Shehata, S. I., Shaarawy, S. S., & Ashry, M. H. H. (2017). Near-miss cases admitted to a maternal intensive care unit, Alexandria, Egypt. *Eastern Mediterranean Health Journal*, 23(10), 694-702. doi:10.26719/2017.23.10.694
122. Yi, H. Y., Jeong, S. Y., Kim, S. H., Kim, Y., Choi, S. J., Oh, S. Y., . . . Kim, J. H. (2018). Indications and characteristics of obstetric patients admitted to the intensive care unit: a 22-year review in a tertiary care center. Obstetrics & Gynecology Science, 61(2), 209-219. doi:10.5468/ogs.2018.61.2.209
123. Yuqi, L., Tan, G., Chengming, S., & Xuri, S. (2017). The ICU Is Becoming a Main Battlefield for Severe Maternal Rescue in China: An 8-Year Single-Center Clinical Experience. *Critical Care Medicine*, 45(11), e1106-e1110. doi:10.1097/ccm.0000000000002597
124. Zhao, Z., Han, S., Yao, G., Li, S., Li, W., Zhao, Y., . . . Han, Y. (2018). Pregnancy-Related ICU Admissions From 2008 to 2016 in China: A First Multicenter Report. Criticsl Care Medicine, 46(10), e1002-e1009. doi:10.1097/ccm.0000000000003355
125. Zorrilla AD, & Segovia MR. (2017). Admission of woman to an Intensive Care Unit during pregnancy or the postpartum period *Revista Nacional (Itagua*), 9(1), 49-60. doi: 10.18004/rdn2017.0009.01.049-060
126. Rudakemwa, A., Cassidy, A. L., & Twagirumugabe, T. (2021). High mortality rate of obstetric critically ill women in Rwanda and its predictability. *BMC Pregnancy and Childbirth*, 21(1), 401-401. doi:10.1186/s12884-021-03882-7
127. Alves, P. P., Costa Volpon, L., & Carmona, F. (2021). Pregnant adolescents admitted to the intensive care unit have better outcomes than pregnant adult women: A retrospective cohort study in Brazil. *International Journal of Gynaecology and Obstetrics*, 155(3), 524-531. doi:10.1002/ijgo.13612
128. Ramlakhan, K. P., Gommers, D., Jacobs, C. E. R. M., Makouri, K., Duvekot, J. J., Reiss, I. K. M., . . . Cornette, J. M. J. (2021). Women of reproductive age in a tertiary intensive care unit: indications, outcome and the impact of pregnancy—a retrospective cohort study. *BMC Womens Health*, 21(1), 1-248. doi:10.1186/s12905-021-01396-0
129. Kumar, K. M., Joshi, A., Saraswat, M., Jose, T., Kapoor, R., Saha, M., & Goyal, B. K. (2021). Near-Miss Incidents in Obstetric Patients Admitted to an Intensive Care Unit of a Tertiary Care Center in Eastern India: A Retrospective Cohort Study. *The Journal of Obstetrics and Gynecology of India*, 1-7. doi:10.1007/s13224-021-01559-x
130. Krawczyk, P., Jastrzebska, A., Lipka, D., & Huras, H. (2021). Pregnancy related and postpartum admissions to intensive care unit in the obstetric tertiary care center - an 8-year retrospective study. *Ginekologia Polska*, 92(8), 575-578. doi:10.5603/GP.a2021.0034
131. Jardine, J., Gurol‐Urganci, I., Harris, T., Hawdon, J., Pasupathy, D., Meulen, J., & Walker, K. (2021). Associations between ethnicity and admission to intensive care among women giving birth: a cohort study. *BJOG: An International Journal of Obstetrics & Gynaecology*. doi:10.1111/1471-0528.16891
132. Gupta, H., Gandotra, N., & Mahajan, R. (2021). Profile of Obstetric Patients in Intensive Care Unit: A Retrospective Study from a Tertiary Care Center in North India. *Indian Journal of Critical Care Medicine*, 25(4), 388-391. doi:10.5005/jp-journals-10071-23775
133. Godeberge, C., Deneux-Tharaux, C., Seco, A., Rossignol, M., Chantry, A. A., & Bonnet, M.-P. (2021). Maternal Intensive Care Unit Admission as an Indicator of Severe Acute Maternal Morbidity: A Population-Based Study. *Anesthesia & Analgesia*. doi:10.1213/ANE.0000000000005578
134. Estrada, P., Jun Ahn, H., & Harvey, S. A. (2021). Racial/Ethnic Disparities in Intensive Care Admissions in a Pregnant and Postpartum Population, Hawai'i, 2012-2017. Public health reports, 333549211021146-333549211021146. doi:10.1177/00333549211021146
135. Simpson, N. B., Shankar-Hari, M., Rowan, K. M., Cecconi, M., von Dadelszen, P., Huning, E. Y. S., . . . Harrison, D. A. (2020). Maternal Risk Modeling in Critical Care—Development of a Multivariable Risk Prediction Model for Death and Prolonged Intensive Care. Critical Care Medicine, 48(5), 663-672. doi:10.1097/CCM.0000000000004223
136. Silva, D. C. E., Lopes, L. G. F., Nunes, M. G. S., Souza, M. A. F. d., Moura, R. N. d., & Medeiros, H. H. d. A. (2020). Perfil de pacientes obstétricas admitidas na unidade de terapia intensiva de um hospital público. *Revista Baiana de Enfermagem*, 34. doi: 10.18471/rbe.v34.35874 Original
137. Rottenstreich, M., Reznick, O., Sela, H. Y., Ioscovich, A., Grisaro Granovsky, S., Weiniger, C. F., & Einav, S. (2020). Severe Maternal Morbidity Cases in Israel in a High-Volume High-Resource Referral Center: A Retrospective Cohort Study. *Israel Medical Association Journal*: *IMAJ*, 22(3), 142-147.
138. Paumier-Galano, E., Suárez-Núñez, E., Capdezuñer-Carcasses, N., Abad-Loyola, P. L., & Navarro-Navarro, V. (2020). Morbilidad materna en la Unidad de Cuidados Intensivos de Baracoa, Guantánamo 2019. *Revista Información Científica*, 99, 233-240.
139. Miglani, U., Pathak, A. P., Laul, P., Sarangi, S., Gandhi, S., Miglani, S., & Laul, A. (2020). A Study of Clinical Profile and Fetomaternal Outcome of Obstetric Patients Admitted to Intensive Care Unit: A Prospective Hospital-based Study. *Indian Journal of Critical Care Medicine*, 24(11), 1071-1076. doi:10.5005/jp-journals-10071-23657
140. Maiden, M. J., Finnis, M. E., Duke, G. J., Huning, E., Crozier, T., Nguyen, N., . . . Pilcher, D. (2020). Obstetric admissions to intensive care units in Australia and New Zealand: a registry-based cohort study. *BJOG: an International Journal of Obstetrics and Gynaecology*, 127(12), 1558-1567. doi:10.1111/1471-0528.16285
141. Khergade, M., Suri, J., Bharti, R., Pandey, D., Bachani, S., & Mittal, P. (2020). Obstetric Early Warning Score for Prognostication of Critically Ill Obstetric Patient. *Indian Journal of Critical Care Medicine*, 24(6), 398-403. doi:10.5005/jp-journals-10071-23453
142. Jayaratnam, S., Jacob-Rodgers, S., & de Costa, C. (2020). Characteristics and preventability of obstetric intensive care unit admissions in Far North Queensland. *The Australian & New Zealand Journal of Obstetrics & Gynaecology*, 60(6), 871-876. doi:10.1111/ajo.13198
143. Hernandez Cabrera, Y., León Ornelas, M. E., Díaz Puebla, J. L., Ocampo Sánchez, A., Rodríguez Márquez, A., & Ruiz Hernández, M. (2020). Caracterización clínica de pacientes con morbilidad materna extremadamente grave y su repercusión perinatal. Cienfuegos 2016-2018. *Medisur*, 18, 789-799.
144. Fong-Pantoja, L. (2020). Morbilidad materna en Unidad de Cuidados Intensivos del Hospital General Docente Orlando Pantoja Tamayo , Contramaestre 2014-2019. *Revista Información Científica*, 99, 20-29.
145. Vargas, M., Marra, A., Buonanno, P., Iacovazzo, C., Schiavone, V., & Servillo, G. (2019). Obstetric Admissions in ICU in a Tertiary Care Center: A 5-Years Retrospective Study. *Indian Journal of Critical Care Medicine*, 23(5), 213-219. doi:10.5005/jp-journals-10071-23163
146. Sailaja, K. B., & Mk, R. (2019). Critically Ill Obstetric Admissions to an Intensive Care Unit: A Prospective Analysis from a Tertiary Care University Hospital in South India. *Indian Journal of Critical Care Medicine*, 23(2), 78-82. doi:10.5005/jp-journals-10071-237121
147. Rossi, R. M., Hall, E., Dufendach, K., & DeFranco, E. A. (2019). Predictive Model of Factors Associated With Maternal Intensive Care Unit Admission. *Obstetrics & Gynecology*, 134(2), 216-224. doi:10.1097/AOG.0000000000003319
148. Prin, M., Kadyaudzu, C., Aagaard, K., & Charles, A. (2019). Obstetric admissions and outcomes in an intensive care unit in Malawi. *International Journal of Obstetric Anesthesia*, 39, 99-104. doi:10.1016/j.ijoa.2019.03.004
149. Oliveira-Neto, A. F., Parpinelli, M. A., Costa, M. L., Souza, R. T., Ribeiro do Valle, C., Sousa, M. H., & Cecatti, J. G. (2019). Prediction of Severe Maternal Outcome Among Pregnant and Puerperal Women in Obstetric ICU. *Critical Care Medicine*, 47(2), e136-e143. doi:10.1097/CCM.0000000000003549
150. Oliveira, S., Filipe, C., Husson, N., Vilhena, I. R., Anastácio, M., Miranda, M., & Devesa, N. (2019). Obstetric Admissions to the Intensive Care Unit: A 18-Year Review in a Portuguese Tertiary Care Centre. *Acta Médica Portuguesa*, 32(11), 693. doi:10.20344/amp.11410
151. Lin, L., Chen, Y.-H., Sun, W., Gong, J.-J., Li, P., Chen, J.-J., . . . Chen, D.-J. (2019). Risk factors of obstetric admissions to the intensive care unit: An 8-year retrospective study. *Medicine*, 98(11), e14835-e14835. doi:10.1097/MD.0000000000014835
152. Fadiloglu, E., Bulut Yuksel, N. D., Unal, C., Ocal, S., Akinci, S. B., Topeli, A., & Beksac, M. S. (2019). Characteristics of obstetric admissions to intensive care unit: APACHE II, SOFA and the Glasgow Coma Scale. *Journal of Perinatal Medicine*, 47(9), 947-957. doi:10.1515/jpm-2019-0125
153. Aoyama, K., Pinto, R., Ray, J. G., Hill, A. D., Scales, D. C., Lapinsky, S. E., . . . Fowler, R. A. (2019). Variability in intensive care unit admission among pregnant and postpartum women in Canada: a nationwide population-based observational study. *Critical Care*, 23(1), 381-381. doi:10.1186/s13054-019-2660-x
154. Chantry, A. A., Monnet, C., Fresson, J., Miller, D., Bonnet, M. P., & Deneux-Tharaux, C. (2021). Repeated maternal ICU admission: Results from a nationwide analysis. *Anaesthesia Critical Care Pain Medicine*, 40(5), 100905. doi:10.1016/j.accpm.2021.100905
155. Panda, S. R., Jain, M., & Jain, S. (2018). Clinical Profile of Obstetric Patients Getting Admitted to ICU in a Tertiary Care Center Having HDU Facility: A Retrospective Analysis. The Journal of Obstetrics and Gynaecology of India, 68(6), 477-481. doi:10.1007/s13224-017-1080-6
156. Ayala Quintanilla, B., P., Pollock, W., McDonald, S., & Taft, A. (2020). Intimate partner violence and severe acute maternal morbidity in the intensive care unit: A case-control study in Peru. Birth, 47(1), 29-38. doi:https://doi.org/10.1111/birt.12461
